# A translational cross-modal control-cost framework for executive breakdown

**DOI:** 10.3389/fnins.2026.1837760

**Published:** 2026-08-06

**Authors:** Yuxin Zhang

**Affiliations:** Independent Researcher, Kaili, Guizhou, China

**Keywords:** biomarker, clinical neuroscience, computational psychiatry, executive dysfunction, executive friction, psychophysiology, task-switching, translational neuroscience

## Abstract

Across neurological and neuropsychiatric conditions, patients often know what they should do yet cannot bring themselves to do it. This dissociation between intact knowledge and failed implementation, which we call the knowing-doing gap, is clinically important but lacks a unified, measurable account. We propose that converting knowledge into action carries a cost, termed *executive friction* (Ψ_*f*_), and that behavior breaks down when this cost exceeds the control resources available to pay it. The proposal is specified at two levels: a measurable factor estimated from observable indicators, and a family of candidate mechanisms in which friction is read either as accumulated control effort or as departure from a habitual default. We model distress as a function of how rapidly friction is rising, how the person appraises the situation, and of executive collapse, which we treat as the point at which control demands outstrip available capacity. The central contribution is practical: a low-cost testing chain that combines behavioral control tasks, shifts in the language of ability (e.g., “can” vs. “cannot”), and autonomic physiology (heart-rate variability and skin conductance), with neural and biochemical measures kept as optional add-ons. Depression, obsessive-compulsive disorder, and Parkinson's disease serve as contrasting test cases with distinct predicted signatures rather than as replacements for existing diagnostic categories. We present five preregistration-ready hypotheses with explicit rejection criteria. No new data are reported.

## 1 Introduction

### 1.1 The knowing–doing gap

Consider three clinical scenarios. A patient with Parkinson's disease can describe, precisely, the sequence of movements required to stand from a chair, yet remains seated, unable to initiate the motor program. A patient with obsessive-compulsive disorder recognizes that her hand-washing compulsion is irrational and disproportionate, yet cannot resist executing it. A patient with major depressive disorder articulates what steps would improve his situation (exercise, social contact, and medication adherence), yet lies immobile, unable to translate knowledge into action.

These scenarios share a striking structural similarity: the agent's representational model of the world and of appropriate action remains intact, while the capacity to *implement* that knowledge is compromised. We refer to this dissociation between knowing and doing as the *knowing–doing gap*. It is among the most clinically significant yet theoretically underspecified phenomena in cognitive neuroscience. Executive function research has cataloged the behavioral signatures of the gap extensively (Miyake et al., 2000; Diamond, 2013; Friedman and Robbins, 2022), and neuroimaging studies have identified associated neural substrates (Stuss and Alexander, 2007; Miller and Cohen, 2001). Yet no existing framework provides a *unified quantitative account* that explains why knowledge and action can decouple, predicts when they will, and specifies how to measure decoupling across behavioral, physiological, neural, and linguistic domains.

The gap itself is not a new observation. Social and health psychology have long studied the “intention–behavior gap,” and the implementation-intention literature shows that specifying when, where, and how one will act measurably improves the translation of goals into behavior, with identifiable physiological correlates (Gollwitzer, 1999; Wieber et al., 2015). In clinical science, the same decoupling recurs across disorders: goal-directed control gives way to habit in obsessive-compulsive disorder (Gillan et al., 2016), initiation fails despite preserved motor knowledge in Parkinson's disease, and intended action stalls in major depressive disorder, motivating transdiagnostic accounts of executive dysfunction (Snyder et al., 2015; McTeague et al., 2017). What remains underrepresented is not the phenomenon but a single, formally defined, measurable *quantity* that ties these scattered descriptions together, explains the gap as one specific failure mode, and yields disorder-differentiating predictions. That quantity is what the present framework proposes.

### 1.2 Limitations of current frameworks

Several theoretical frameworks each identify a real piece of the knowing–doing gap, but none supplies the missing element: a single cost quantity that is measurable across behavior, physiology, and language simultaneously. We review five, noting for each what it captures and what it leaves out.

**Cognitive load theory** (Sweller, 1988; Sweller et al., 2019) evidenced the demands placed on working memory during learning and task execution. While influential in educational psychology, it remains a purely behavioral construct without physiological or neural grounding, and it does not address the *maintenance cost* of sustaining a selected state over time against competing defaults.

**The Free Energy Principle** (FEP; Friston, 2010) provides an elegant variational framework in which organisms minimize prediction error. The complexity term in variational free energy (*D*_*KL*_[*Q* ∥ *P*]) captures part of what we mean by friction: the cost of a model deviates from priors. However, FEP treats this cost as something to be *minimized*, and does not provide a framework for situations in which sustained high deviation cost is adaptive (creative problem-solving, or ethical resistance to default heuristics) or for the *failure mode* in which deviation cost overwhelms the system's capacity.

**Integrated Information Theory** (IIT; Tononi et al., 2016) offers a scalar measure of consciousness (Φ) based on the irreducibility of cause-effect structures. While Φ captures integration; it does not address the *dynamic cost* of maintaining integrated states or the transition costs between states. It provides a snapshot measure, not a cost function.

**Allostatic load** (McEwen, 1998; McEwen and Stellar, 1993) captures the cumulative physiological cost of chronic stress adaptation, but operates at too coarse a temporal grain to explain moment-to-moment executive breakdowns, and lacks a formal connection to information-theoretic quantities.

**Classical ego depletion models** conceptualize self-control as a limited resource (Baumeister et al., 1998), but glucose-specific substrate interpretations have received substantial methodological criticism, weak meta-analytic support, and replication concerns (Hagger et al., 2016; Vadillo et al., 2016). This motivates treating metabolic readouts as testable correlates rather than fixed assumptions.

The critical gap is this: none of these frameworks provides a *single formally defined quantity* that (a) is grounded in information-theoretic and energetically interpretable resource-budget terms, (b) generates measurable predictions across behavioral, physiological, neural, and linguistic domains simultaneously, (c) explains the knowing–doing gap as a specific failure mode, and (d) produces disorder-specific clinical predictions. The construct proposed below is designed to fill exactly this gap. It connects aspects of, and extends selected cost-related aspects of, the frameworks above rather than competing with or replacing them: it relates to cognitive load on the behavioral side, to allostatic load on the physiological side, and to variational complexity on the information-theoretic side, while adding the cross-modal and dissociation predictions that none of them makes (developed in Section 6.2).

### 1.3 Contribution and scope

In plain terms, this study proposes a single idea and then develops it. The idea is that turning knowledge into action has a cost, which we call *executive friction*, and that when this cost grows too high, three things follow. Behavior can break down abruptly rather than gradually. The language people use about action can shift measurably, tilting from “can” toward “cannot.” Furthermore, behavioral, physiological, and linguistic measures should move together, because they reflect one underlying cost rather than three unrelated problems. The framework is deliberately designed to be tested with low-cost, widely available methods, using depression, obsessive-compulsive disorder, and Parkinson's disease as contrasting clinical test cases. Everything that follows, including the formal notation, is in service of making that one idea measurable and falsifiable.

More formally, this article is positioned as a translational neuroscience hypothesis document on executive breakdown under resource-bounded action selection. We introduce *executive friction* (Ψ_*f*_) as a latent cross-modal control-cost factor and evaluate it as a candidate bridge between computational theory, clinically accessible biomarkers, and disorder-differentiating predictions. The Ψ_*f*_ framework makes three specific predictions that are jointly stronger than prior accounts: a non-linear critical-load transition (ρ_*c*_), context-sensitive modal-language shifts (μ_sem_), and convergence of behavioral-linguistic-physiological indicators onto a single latent factor. The core validation chain is intentionally low-cost and scalable: behavioral control tasks, adjusted modal-language scores, and autonomic physiology (heart rate variability [HRV]/skin conductance response [SCR], with cortisol optional). Higher cost neural and biochemical measures are treated as optional adjudication layers rather than prerequisites. Major depressive disorder, obsessive-compulsive disorder, and Parkinson's disease are included as boundary use cases for stress-testing parameter drift and dissociation patterns, not as attempts to replace disease-specific pathophysiology. The main text is written in standard hierarchical-control and predictive-processing language, and Appendix A is kept strictly as optional notation support rather than as an entry point to any larger philosophical system.

This is a theoretical and computational contribution aimed at empirical translation. No new data are reported. Consistent with the *Frontiers in Neuroscience* Hypothesis and Theory format, the article advances a testable model within a specific area of investigation (the knowing-doing gap in executive dysfunction) and provides a measurement strategy, falsification rules, and clinically tractable protocols for future adjudication.

### 1.4 Standard control terms used in this article

To reduce interpretation burden, the main text uses established control-theoretic and predictive-processing language throughout:

- Ψ_*f*_: estimable control/execution cost (latent cross-modal factor), termed “executive friction.”
- *P*_sel_: available control budget (selection capacity proxy).
- Candidate policy space: the set of task-relevant options or policies available to the agent.
- Active state: the currently implemented percept-action or task-control state.
- Slow constraints: habits, priors, hyperpriors, and trait-like parameters that shape future selections.
- Resource-bounded selection/gating mechanism: the control process that maps candidate policies into active execution under finite neural and bodily resources.

Readers who prefer compact symbols can consult Appendix A, but the argument of the article does not depend on any special notation.

## 2. Hierarchical control framework for executive friction

### 2.1 A Minimal hierarchical control scaffold

The framework developed here can be read entirely within standard hierarchical control language. The three-part decomposition is a pragmatic level-of-description choice rather than a new ontology. Readers do not need to adopt any broader philosophical framework to evaluate Ψ_*f*_: the same equations are intended to be compatible with working-memory gating accounts, resource-rational control formulations, and active-inference/predictive-coding control loops (Miller and Cohen, 2001; Frank et al., 2004; Friston, 2010; Shenhav et al., 2017).

#### 2.1.1 Candidate policy space

This denotes the task-relevant set of candidate states or strategies available to the agent at a given time.

#### 2.1.2 Active state

This denotes the perceptual interpretation and action policy currently being implemented.

#### 2.1.3 Slow constraints

These are the slower variables that shape future selections, including habits, model priors, hyperpriors, and trait-like control limits.

These layers are linked by a **resource-bounded selection mechanism**, which maps candidate policies into executed states:

$$\begin{array}{l} x_{\text{a}\text{c}\text{t}}\left(t\right)={\text{S}\text{e}\text{l}\text{e}\text{c}\text{t}}_{\theta }\left[\Pi \left(t\right)\right] \end{array}$$

where Π(*t*) denotes the candidate policy space, *x*_act_(*t*) refers to the active state, and θ represents the finite embodiment parameters (e.g., network weights, neuromodulatory state, and metabolic constraints). The subscript θ emphasizes resource-bounded selection.

Slow constraints update through stabilization:

$$\begin{array}{l} z\left(t+1\right)=\text{S}\text{t}\text{a}\text{b}\text{i}\text{l}\text{i}\text{z}\text{e}\left(z\left(t\right),x_{\text{a}\text{c}\text{t}}\left(t+1\right)\right)\; \end{array}$$

Within this architecture, the key claim is that maintaining or switching the active state relative to a baseline policy trajectory incurs a measurable control cost, termed executive friction (Ψ_*f*_), that constrains executive function.

### 2.2 Executive friction (Ψ_f_): formal definition

We define executive friction through a two-level specification, and the distinction between the two levels is important, so we state it up front. **Definition 1A specifies how** Ψ_*f*_ **is estimated from observable data**: it is the operational, measurement-level definition, and it is what the empirical program actually fits. **Definition 1B offers candidate mechanistic interpretations** of what could generate that estimated quantity: these are competing theoretical models, tested against each other, and none of them is required in order to estimate Ψ_*f*_ in the first place. A reader who accepts only Definition 1A can still use the entire measurement and clinical program that follows. Throughout, the term “executive friction” denotes the cross-layer maintenance and switching costs that an agent incurs when sustaining or reconfiguring an active control state relative to baseline defaults.

#### 2.2.1 Definition 1A (level-0 empirical latent variable)

At the measurement level, Ψ_*f*_ is a latent factor jointly loaded by behavioral, linguistic, and physiological indicators (with neural and biochemical indicators used as expansion modalities). This is the definition the analysis estimates directly:

$$\begin{array}{l} y_{k}=\lambda_{k}{\text{Ψ}}_{f}+\varepsilon_{k},\;k=1,\ldots ,K \end{array}$$ (3a)

where *y*_*k*_ are standardized observed indicators, λ_*k*_ are factor loadings, and ε_*k*_ are residuals. This is the estimand used in structural equation modeling (SEM) and confirmatory factor analysis (CFA).

#### 2.2.2 Definition 1B (level-1 mechanistic control cost)

Definition 1B proposes what could physically generate the latent factor of 1A. We give two candidate mechanisms and treat them as rival models to be compared on data, not as established facts. Let the state dynamics be

$$\begin{array}{lll} ẋ\left(t\right) & = & f_{\theta }\left(x,t\right)+B_{\theta }\left(x,t\right)u\left(t\right)+\xi \left(t\right),\\ {ẋ}_{0}\left(t\right) & = & f_{\theta }\left(x,t\right)+\xi \left(t\right)\;\text{w}\text{h}\text{e}\text{n}\;u\left(t\right)=0 \end{array}$$ (3b)

where *x*(*t*) is the system state, *f*_θ_ is the uncontrolled (drift) dynamics, *u*(*t*) is the control input, *B*_θ_ maps control input onto the state, and ξ(*t*) is a zero-mean stochastic noise term representing intrinsic neural and measurement variability (e.g., a Wiener increment). Setting *u*(*t*) = 0 gives ẋ_0_, the no-control *baseline drift* trajectory, that is, what the system would do if no control effort were spent.

The first candidate reads friction as **accumulated control effort**: the running total of how hard the controller has to push to keep the state on course rather than letting it drift,

$$\begin{array}{l} {\text{Ψ}}_{f}^{\text{c}\text{t}\text{r}\text{l}}\left(T\right)=\int_{0}^{T}u{\left(t\right)}^{⊤}Ru\left(t\right)dt,\;R≻0 \end{array}$$

where *R* ≻ 0 is a positive-definite cost matrix weighting the effort. The second candidate reads friction instead as a **departure from the habitual default policy**, measured as the information-geometric distance between the current control distribution *q*_*t*_ and the baseline-policy distribution $q_{t}^{0}$, accumulated over time,

$$\begin{array}{l} {\text{Ψ}}_{f}^{\text{i}\text{n}\text{f}\text{o}}\left(T\right)=\int_{0}^{T}D_{KL}\left(q_{t}∥q_{t}^{0}\right)dt \end{array}$$ (3d)

where *D*_*KL*_ is the Kullback–Leibler (KL) divergence and $q_{t}^{0}$ denotes the baseline-policy distribution. Intuitively, Equation 3c charges for *how much force* the controller applies, whereas Equation 3d charges for *how far* the chosen policy strays from habit; the two can diverge, which is what makes them empirically separable. Under regularity conditions (e.g., locally smooth trajectories and second-order KL expansion in exponential-family neighborhoods), the KL form admits a Fisher-metric approximation (Amari, 2016):

$$\begin{array}{l} {\text{Ψ}}_{f}^{\text{K}\text{L}-2\text{n}\text{d}}\left(T\right)\approx \int_{0}^{T}\dot{\vartheta }{\left(t\right)}^{⊤}g_{F}\left(\vartheta \right)\dot{\vartheta }\left(t\right)dt \end{array}$$ (3d')

In this article, Ψ_*f*_ denotes the Level-0 latent construct, while Equations 3c, 3d, 3d' are treated as competing/related mechanism models to be compared empirically rather than universally equivalent identities.

#### 2.2.3 Mechanism-model comparison plan

Equation 3c is treated as a control-energy model best matched to trial-level demand manipulations (e.g., switch/inhibition load), whereas Equations 3d, 3d' are treated as distributional-deviation models for belief-drift or habit-override regimes. In empirical programs, matched hierarchical models will be fit to the same outcomes and compared with out-of-sample criteria (leave-one-out cross-validation [LOO] and the Widely Applicable Information Criterion [WAIC]), with Bayes factors where model regularity allows. Pre-registered interpretation is: if the Equation 3c-family wins, friction is operationally summarized as control effort; if Equations 3d, 3d' win, friction is better summarized as information-geometric deviation; if no mechanism family dominates, Equation 3a remains the estimand and mechanism equations are treated as context-specific modules.

Definitions 1A and 1B specify executive friction itself. We now turn to the *distress* that accompanies it, which is a separate quantity: an agent can carry high friction quietly, and what hurts is friction that is rising quickly and is appraised as threatening. Definition 2 captures this. We write it as a “hazard function,” meaning the momentary risk that distress crosses into a felt state, and this risk rises with the rate of change of friction and with the person's appraisal of the situation.

#### 2.2.4 Definition 2 (distress hazard family)

Subjective distress is modeled as a hazard-family function of *smoothed* friction change and appraisal state:

$$\begin{array}{l} h_{\text{d}\text{i}\text{s}\text{t}\text{r}\text{e}\text{s}\text{s}}\left(t\right)=\sigma \left(a\frac{d{\tilde{\text{Ψ}}}_{f}^{\left(\tau \right)}\left(t\right)}{dt}+c\text{A}\text{p}\text{p}\text{r}\text{a}\text{i}\text{s}\text{a}\text{l}\left(t\right)-b\right) \end{array}$$

where ${\tilde{\text{Ψ}}}_{f}^{\left(\tau \right)}$ is a smoothed friction trajectory (window τ), σ(·) is a monotone link (e.g., logistic), and Appraisal captures contextual evaluation (Gaab et al., 2005). In the local linear limit with approximately constant appraisal, Equation 4 reduces to the familiar approximation *distress* ∝ *dΨ*_*f*_/*dt*. This preserves the core intuition that rapidly increasing friction destabilizes while avoiding the stronger, less defensible claim that all pain is driven solely by raw derivatives.

For preregistration, τ is treated as a sensitivity parameter (default grid: 10, 30, and 60 s, or trial-window analogs), and Appraisal is measured block-wise on a 0–100 challenge/threat scale immediately after each block to reduce retrospective distortion.

Having defined friction (Definition 1) and the distress that tracks it (Definition 2), we now state the budget that decides whether the system holds together at all. The intuition is an accounting one: an agent has a finite control budget, friction and noise draw on it, and whatever remains determines whether the current task-state can be kept orderly or begins to fall apart.

#### 2.2.5 Definition 3 (selection budget inequality; thermodynamic-inspired)

The rate of change of macroscopic task-state order *q*(*x*_act_), operationalizable as mutual information density, topological invariants, or compression ratio, is bounded by:

$$\begin{array}{l} \frac{dq}{dt}\leq \alpha \cdot P_{\text{s}\text{e}\text{l}}\left(t\right)-\beta \cdot {\text{Ψ}}_{f}\left(x_{\text{a}\text{c}\text{t}};\theta \right)-\gamma \cdot S_{\text{n}\text{o}\text{i}\text{s}\text{e}} \end{array}$$

where *P*_sel_(*t*) is the *selection power* (the net control budget available for maintaining and extending the current active state), Ψ_*f*_ is the friction cost, *S*_noise_ is the environmental noise entropy, and α, β, γ > 0 are coupling constants.

This inequality is the master budget constraint of the present framework. It states that an agent's capacity to maintain or increase order in its active control state is bounded by available selection power minus friction and noise costs. In early-phase experiments, *q*(*x*_act_) is treated as a boundary variable rather than a mandatory primary endpoint; optional operationalizations include compression ratio, entropy-rate surrogates, or task-state stability metrics. When *P*_sel_ < β · Ψ_*f*_ + γ · *S*_noise_, the system enters *order collapse* (*dq*/*dt* < 0), manifesting as cognitive disorganization or executive failure.

#### 2.2.6 Definition 4 (parameter dynamics)

The embodiment parameters θ evolve under three competing pressures: error-driven learning, friction-potential descent, and homeostatic recoil. Because none of the five phase-1/2 hypotheses directly test these slow dynamics (which operate over weeks to months rather than within-session timescales), the formal equation is presented in Appendix B. For present purposes, the key implication is that therapeutic interventions can be modeled as modifying either the learning rate, the friction landscape, or the homeostatic set point, distinctions that become empirically testable only in longitudinal treatment designs beyond the scope of the current validation roadmap.

### 2.3 The knowing–doing gap as bandwidth saturation

The knowing–doing gap emerges naturally from the selection-budget inequality (Equation 5). Consider an agent whose slow constraints and learned model correctly specify the appropriate action, such as a patient who knows she should exercise or a surgeon who knows the next step in the procedure. The knowledge representation is intact.

Throughout this article, **selection power** (*P*_sel_) is the primary term; “bandwidth” is used only as an informal synonym for residual selection capacity.

However, *implementing* this knowledge requires converting it into an active action state through a resource-bounded selection or gating process. This conversion consumes selection power *P*_sel_ and incurs executive friction Ψ_*f*_. When the agent is already operating under high baseline friction (due to chronic stress, neurodegeneration, or pathological parameter configurations), the remaining selection power may be insufficient to execute the known action.

Formally, the agent enters the knowing–doing gap when:

$$\begin{array}{l} P_{\text{s}\text{e}\text{l}}^{\text{r}\text{e}\text{s}\text{i}\text{d}\text{u}\text{a}\text{l}}=P_{\text{s}\text{e}\text{l}}-\beta \cdot {\text{Ψ}}_{f}^{\text{b}\text{a}\text{s}\text{e}\text{l}\text{i}\text{n}\text{e}}<{\text{Ψ}}_{f}^{\text{a}\text{c}\text{t}\text{i}\text{o}\text{n}} \end{array}$$

where ${\text{Ψ}}_{f}^{\text{b}\text{a}\text{s}\text{e}\text{l}\text{i}\text{n}\text{e}}$ is the friction cost of maintaining the current state, and ${\text{Ψ}}_{f}^{\text{a}\text{c}\text{t}\text{i}\text{o}\text{n}}$ is the additional friction cost of initiating the target action. The agent's learned model is intact (she “knows”), but the selection/gating system lacks the residual capacity to execute (she “can't do”).

This formulation makes a specific prediction: the knowing–doing gap should be *non-linearly* related to baseline friction load. At low ${\text{Ψ}}_{f}^{\text{b}\text{a}\text{s}\text{e}\text{l}\text{i}\text{n}\text{e}}$, adding task demands produces proportional increases in task-switching cost (linear regime). As ${\text{Ψ}}_{f}^{\text{b}\text{a}\text{s}\text{e}\text{l}\text{i}\text{n}\text{e}}$ approaches *P*_sel_, small additional demands produce steep performance collapse (saturation regime).

To parameterize this as a fit-ready critical phenomenon, define normalized control load:

$$\begin{array}{l} \rho \left(t\right)=\frac{\beta \cdot {\text{Ψ}}_{f}^{\text{b}\text{a}\text{s}\text{e}\text{l}\text{i}\text{n}\text{e}}\left(t\right)}{P_{\text{s}\text{e}\text{l}}\left(t\right)} \end{array}$$

and model switch cost with a changepoint:

$$\begin{array}{l} C_{\text{s}\text{w}\text{i}\text{t}\text{c}\text{h}}\left(\rho \right)=c_{0}+c_{1}\rho +c_{2}{\left(\rho -\rho_{c}\right)}_{+}^{2},\;{\left(x\right)}_{+}=\text{m}\text{a}\text{x}\left(x,0\right) \end{array}$$

where ρ_*c*_ is the estimated critical point. The model predicts a stable linear region for ρ < ρ_*c*_ and accelerated cost growth for ρ ≥ ρ_*c*_, with ρ_*c*_ expected in a high-load band near resource saturation. Empirically, ρ_*c*_ can be estimated via segmented regression, threshold mixed models, or Bayesian changepoint inference.

### 2.4 Relationship to predictive processing and free energy minimization

Why relate the proposal to the Free Energy Principle at all? Two reasons. First, FEP is currently the most influential formal account of neural cost, so showing that executive friction connects to it demonstrates that Ψ_*f*_ is not an *ad hoc* quantity but a recognizable generalization of an established one. Second, the connection sharpens exactly where our claim departs from FEP, and that departure is the clinically load-bearing part of the argument. Concretely, Ψ_*f*_ bears a formal relationship to the complexity term in variational free energy. In the FEP framework, the variational free energy *F* decomposes as expressed in the following:

$$\begin{array}{l} F=\underset{\text{Complexity}}{\underset{\underset{\;}{\underset{︸}{\;}}}{D_{KL}[Q(\theta )∥P(\theta )]}}-\underset{\text{Accuracy}}{\underset{\underset{\;}{\underset{︸}{\;}}}{E_{Q}[\text{ln}P(\text{data}|\theta )]}} \end{array}$$

The complexity term *D*_*KL*_[*Q* ∥ *P*] penalizes posteriors that deviate from priors, formally analogous to the information-geometric friction form in Equation 3d. In this reading, Ψ_*f*_ generalizes complexity cost by (a) accumulating over time, (b) applying to executed percept-action control rather than beliefs alone, and (c) linking to energetic budgets through Equation 5.

The critical distinction between the present framework and the standard FEP is in the normative implication. FEP implies that systems *should* minimize free energy and, by extension, minimize the complexity cost. The present framework allows that some states *require* sustained high Ψ_*f*_: creative insight that resists premature closure, ethical choices that resist default heuristics, or grief that maintains connection to the departed. In these cases, high Ψ_*f*_ is not pathological but constitutive of the valued state. Pathology arises not from high Ψ_*f*_ *per se*, but from *unsustainable* Ψ_*f*_, that is, when the cost chronically exceeds the agent's selection power budget.

This distinction has immediate clinical relevance: the therapeutic goal is not to eliminate Ψ_*f*_ (which would eliminate agency) but to restore the balance between Ψ_*f*_ and *P*_sel_, either by reducing unnecessary friction or by expanding selection power capacity.

### 2.5 Dimensionalization and identifiability (empirical budget form)

Equation 5 is used in this study as an **empirically identifiable statistical budget model**, not as a closed-form physical law with fixed SI units. For phase-1/2 inference, we estimate all terms in standardized space:

$$\begin{array}{l} \frac{d\tilde{q}}{dt}\leq w_{0}+\alpha {\tilde{P}}_{\text{s}\text{e}\text{l}}-\beta {\tilde{\text{Ψ}}}_{f}-\gamma {\tilde{S}}_{\text{n}\text{o}\text{i}\text{s}\text{e}}+\varepsilon_{t} \end{array}$$ (5z)

where tildes denote z-scored quantities, and α, β, *and γ* are regression weights to be estimated from data (not universal physical constants). In this form, $\tilde{q}$ is a dimensionless state-stability/order index, and all predictors are unitless standardized indicators. Minimal proxy anchoring follows Table 1: ${\tilde{P}}_{\text{s}\text{e}\text{l}}$ from HRV/SCR recovery indicators, ${\tilde{\text{Ψ}}}_{f}$ from latent-factor estimation over behavioral-linguistic-physiological indicators, and ${\tilde{S}}_{\text{n}\text{o}\text{i}\text{s}\text{e}}$ from task conflict/entropy manipulations.

**Table 1:** Equation-to-proxy mapping for core and expansion layers.

Model term	Functional interpretation	Core measurement mapping	Expansion mapping
	Available control budget	HRV recovery slope, SCR recovery profile, diurnal cortisol slope	PCI and network-level complexity indices
	Latent control cost	Task-switch cost, Stroop interference, perseverative errors, $\mu_{\text{s}\text{e}\text{m}}^{\text{a}\text{d}\text{j}}$	ROS markers, Fisher-geometry metrics
*S*_noise_ in Equation 5	Environmental/task uncertainty load	Conflict level manipulations, distractor entropy, and prompt variability controls	Ecological noise paradigms
*q*(*x*_act_) in Equation 5	Task-state order/stability	q^*^ from error entropy and RT variability (Equation 5q)	Compression-ratio or sequence-complexity alternatives
Appraisal(*t*) in Equation 4	Contextual threat/challenge evaluation	Trial/block-wise self-report rating (0-100 challenge/threat scale)	Multi-item stress appraisal inventories
*u*(*t*) in Equation 3c	Momentary control input effort	Trial-wise inhibitory/control demand (e.g., incongruent Stroop, switch trials, rule override trials)	Neural control-energy surrogates from EEG/TMS paradigms
ρ and ρ^*^ in Equations 7a, 7c	Normalized friction load	Primary: ρ^*^ (score-based); sensitivity: ratio-based ρ	Cross-modal validation with neural/biochemical layers

For confirmatory phase-1 analyses, we operationalize *q*(*x*_act_) with a minimal observable index:

$$\begin{array}{l} q^{*}\left(t\right)=\frac{1}{2}z\left(-H_{\text{e}\text{r}\text{r}\text{o}\text{r},w}\left(t\right)\right)+\frac{1}{2}z\left(-CV_{\text{R}\text{T},w}\left(t\right)\right)\; \end{array}$$

where *H*_error, *w*_ is windowed response-error entropy and *CV*_RT, *w*_ is windowed reaction-time coefficient of variation. Response-error entropy is computed as follows: within a sliding window of *w* consecutive trials, each response is assigned to an outcome category (e.g., correct, commission error, omission error), and the empirical probabilities *p*_*c*_ of these categories are estimated, and their Shannon entropy $H_{\text{e}\text{r}\text{r}\text{o}\text{r},w}=-\underset{c}{\sum }p_{c}\log p_{c}$ is taken. Low entropy means responses are consistent and predictable (a stable control state); high entropy means outcomes are scattered across categories (a disorganized one). Because both terms in q^*^ are sign-flipped and z-scored, higher q^*^ indicates greater local task-state stability, that is, both more predictable errors and less variable reaction times.

Interpretively, q^*^ captures a minimal “order/stability” surrogate: stable control states should show lower local error unpredictability and lower RT dispersion. To separate this from generic arousal/fatigue accounts, phase-1 models include baseline arousal and block-order covariates; if q^*^ variance is explained by arousal/fatigue alone, while budget terms contribute no directional signal; the Equation 5 interpretation is weakened. The confirmatory phase-1 test for Equation 5 is directional and statistical: whether ${\tilde{P}}_{\text{s}\text{e}\text{l}}$, ${\tilde{\text{Ψ}}}_{f}$, and ${\tilde{S}}_{\text{n}\text{o}\text{i}\text{s}\text{e}}$ jointly predict dq^*^/*dt* with expected signs, rather than whether Equation 5 is a literal thermodynamic identity.

**Figure 1:**
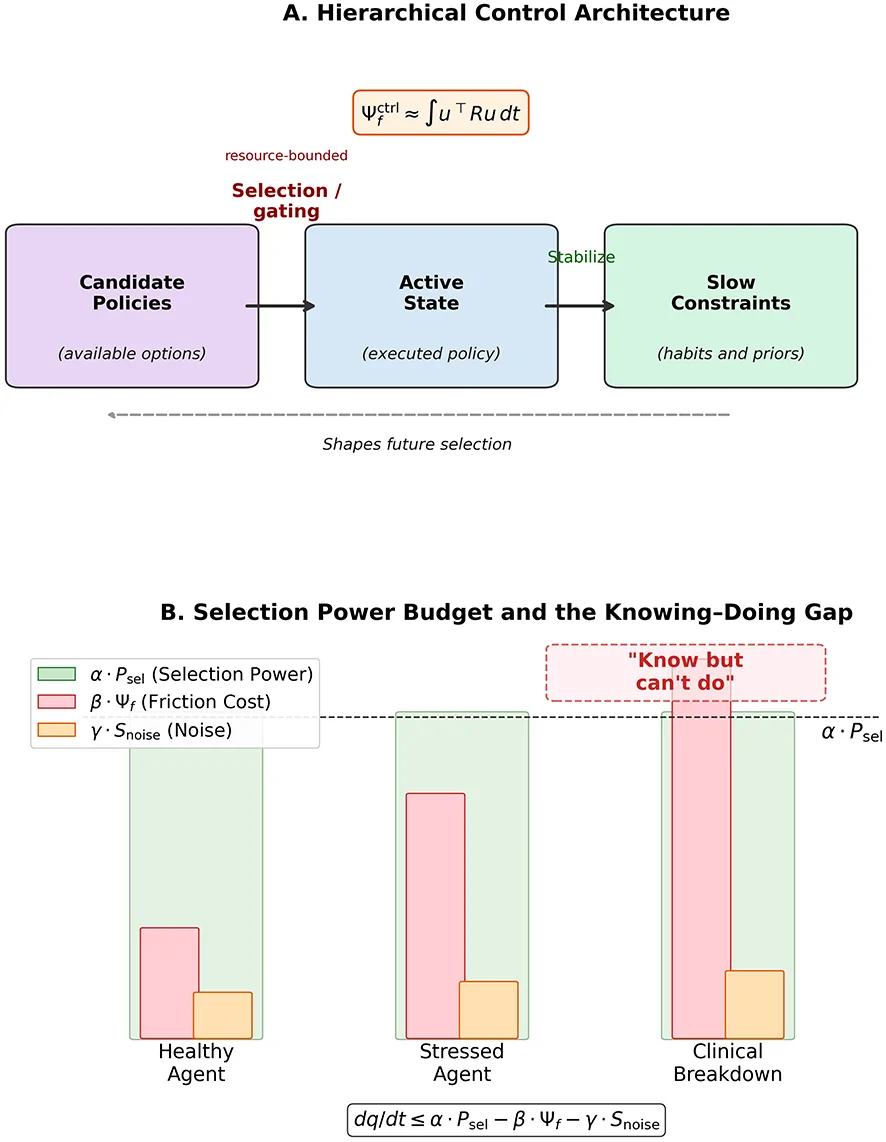
*Hierarchical control architecture and selection power budget*. **(A)** Three-part control scaffold: candidate policy space, active state, and slow constraints, linked by a resource-bounded selection/gating mechanism. Dashed feedback arrow indicates how slow constraints shape future selection. **(B)** Selection-budget diagram illustrating how the “knowing-doing gap” emerges when the red bar (β · Ψ_*f*_) plus orange bar (γ · *S*_noise_) exceeds the green bar (α · *P*_sel_), progressing from healthy function through stressed operation to clinical breakdown. Final artwork should keep these exact symbol labels for one-to-one correspondence with Equation 5.

For changepoint testing, we retain the theoretical ratio in Equation 7a and additionally define a phase-1 robust approximation:

$$\begin{array}{l} \rho^{*}\left(t\right)=z\left({\text{Ψ}}_{f}^{\text{b}\text{a}\text{s}\text{e}\text{l}\text{i}\text{n}\text{e},\;\text{l}\text{a}\text{t}\text{e}\text{n}\text{t}}\left(t\right)\right)-z\left(P_{\text{s}\text{e}\text{l}}^{\text{p}\text{r}\text{o}\text{x}\text{y}}\left(t\right)\right)\; \end{array}$$

which avoids instability of ratio estimators in low-denominator regions. Both forms express the same thing: how close friction has come to exhausting the capacity for control. In the ratio form (Equation 7a), this is friction divided by capacity; in the difference form (Equation 7c) it is standardized friction minus standardized capacity. The *critical point* ρ_*c*_ (or $\rho_{c}^{*}$) is the value of this quantity at which the system's behavior changes character, marking the boundary between the regime where added demand raises switch cost gently and roughly linearly, and the regime where the residual budget is nearly spent, and small additional demands trigger steep collapse. It is a statistical changepoint estimated from data, not a claim about a universal thermodynamic constant (Figure 1).

## 3 Operationalization: four proxy classes for Ψ_f_

A theoretical construct is scientifically useful only to the extent that it can be measured. We now derive four classes of proxy measures for Ψ_*f*_, each grounded in established measurement paradigms but unified under a common latent-variable interpretation. The central claim of this section is that Ψ_*f*_ is not reducible to any single proxy but manifests as a correlated pattern across behavioral, physiological, neural, and linguistic indicators.

### 3.1 Validation-first prioritization

To maximize empirical tractability, this study adopts a staged validation strategy. The **core validation chain** uses low-cost modalities that can be replicated broadly: behavioral control-cost tasks, the linguistic modal-ratio probe (μ_sem_), and low-cost physiology (HRV and SCR; cortisol optional). High-cost markers (reactive oxygen species [ROS] assays, Perturbational Complexity Index [PCI], Fisher-geometry readouts, and theta-gamma coupling) are treated as **expansion layers** for later-phase adjudication rather than prerequisites for first-pass falsification. Failure of expansion markers to add value does not invalidate the core-chain model.

For transparency, we explicitly map model terms to measurable proxies:

### 3.2 Behavioral proxies

#### 3.2.1 Task-switching cost

When an agent must reconfigure the active control state to serve a new task rule, the reconfiguration incurs executive friction proportional to the distance between the current and target control configurations. The classical task-switching cost, namely the increase in reaction time and error rate on switch trials relative to repeat trials (Monsell, 2003; Kiesel et al., 2010), directly indexes this reconfiguration friction. Computational accounts further decompose this cost into reconfiguration and interference-control components (Yeung and Monsell, 2003; Brown et al., 2007), supporting model-based estimation of behavioral friction. We predict that task-switching cost scales with Ψ_*f*_ across individuals and within individuals across conditions.

#### 3.2.2 Perseverative errors

In the Wisconsin Card Sorting Test (WCST; Berg, 1948) and related paradigms, perseverative errors reflect the agent's failure to abandon a high-friction configuration: the previous sorting rule has become deeply anchored (high ${\text{Ψ}}_{f}^{\text{s}\text{w}\text{i}\text{t}\text{c}\text{h}}$) and the system cannot pay the switching cost. The number of perseverative errors indexes the *viscosity* η of the parameter space: η ∝ ∂Ψ_*f*_/∂θ.

#### 3.2.3 Stroop interference

The Stroop effect (Stroop, 1935) measures the friction cost of suppressing a default selection (word reading) in favor of a non-default selection (color naming). The Stroop interference ratio (incongruent RT/congruent RT) provides a normalized behavioral index of the friction differential between default and non-default selections.

#### 3.2.4 Temporal discounting

Steep temporal discounting (the preference for smaller-sooner over larger-later rewards) reflects the high friction cost of maintaining a non-default, future-oriented active control state (Bickel et al., 2012). The temporal discounting rate *k* in the hyperbolic model (*V* = *A*/[1 + *kD*]) can be interpreted as a proxy for the friction cost of temporal extension of the selection horizon. This interpretation is consistent with effort-based decision frameworks that treat control allocation as a cost-benefit computation (Westbrook and Braver, 2015).

#### 3.2.5 Composite score

We propose a composite behavioral Ψ_*f*_ score computed as the average of standardized (Z-scored) values across: (a) mean task-switching cost, (b) WCST perseverative error proportion, (c) Stroop interference ratio, and (d) temporal discounting rate.

### 3.3 Physiological proxies (core and expansion)

#### 3.3.1 Heart rate variability (HRV) recovery

Vagal-mediated HRV reflects the capacity of the autonomic nervous system to regulate arousal, a physiological substrate of selection power *P*_sel_. The neurovisceral integration model (Thayer and Lane, 2009) posits that high resting HRV reflects flexible autonomic control, whereas reduced HRV reflects rigidity. We predict that *HRV recovery slope* (the rate at which HRV returns to baseline following a stressor) indexes the system's capacity to dissipate Ψ_*f*_. Slow recovery indicates sustained high friction; rapid recovery indicates efficient friction resolution.

#### 3.3.2 Cortisol circadian rhythm

The diurnal cortisol curve (cortisol awakening response [CAR] and diurnal slope) reflects the integrity of the hypothalamic-pituitary-adrenal axis. Chronic high Ψ_*f*_ is predicted to flatten the cortisol curve (reducing the morning peak and elevating the evening nadir), reflecting exhaustion of the neuroendocrine substrate that supports selection power (Adam et al., 2017). We propose the cortisol awakening response (CAR) magnitude and the diurnal cortisol slope (DCS) as complementary physiological proxies.

#### 3.3.3 Skin conductance response (SCR)

Phasic SCR amplitude during decision-making tasks reflects momentary spikes in autonomic arousal associated with conflict and uncertainty, that is, acute increases in *dΨ*_*f*_/*dt*. SCR peak amplitude during high-conflict choices (e.g., the Iowa Gambling Task; Bechara et al., 1994) provides a real-time physiological index of the hazard function (Equation 4). Importantly, SCR is treated here as an *acute-state* readout, most appropriate for within-task or cue-locked friction peaks. It is not assumed to track slow, chronic friction accumulation on its own; for chronic-load interpretations (e.g., in major depressive disorder [MDD]), SCR is therefore read alongside slower measures such as HRV recovery, cortisol slope, and trait-level behavioral composites.

#### 3.3.4 Reactive oxygen species (ROS) as a biochemical signature (expansion layer)

We propose a link between Ψ_*f*_ and oxidative stress for later-phase testing. The sustained energetic expenditure required to maintain high-friction states should produce elevated mitochondrial activity and, consequently, elevated ROS production. We formalize this as follows:

$$\begin{array}{l} \frac{d\left[\text{R}\text{O}\text{S}\right]}{dt}=\alpha \cdot {\text{Ψ}}_{f}\left(\text{c}\text{o}\text{n}\text{t}\text{r}\text{o}\text{l}\;\text{a}\text{l}\text{l}\text{o}\text{c}\text{a}\text{t}\text{i}\text{o}\text{n}\right)-\beta \cdot \text{C}\text{l}\text{e}\text{a}\text{r}\text{a}\text{n}\text{c}\text{e}\left(\theta_{\text{b}\text{o}\text{d}\text{y}}\right) \end{array}$$

where α is the friction-to-oxidative-load coupling coefficient, and β · *Clearance* represents antioxidant clearance capacity. Equation 9 is a simplified coupling model: the linear coupling α · Ψ_*f*_ abstracts over multiple intervening biochemical steps (sustained neural metabolic demand → mitochondrial electron-transport load → superoxide production). In empirical use, α is a regression coefficient estimated from data, not a fixed biophysical constant; future mechanistic elaboration may replace the linear term with a saturating or threshold function. Measurable ROS biomarkers include the reduced glutathione (GSH) to oxidized glutathione (GSSG) ratio, malondialdehyde (MDA), 8-hydroxy-2′-deoxyguanosine (8-OHdG), and superoxide dismutase (SOD) activity. This is deliberately positioned as a phase-3 extension after core low-cost validation succeeds.

#### 3.3.5 Falsification condition for ROS coupling

If experimentally induced high-Ψ_*f*_ tasks (e.g., sustained task-switching under time pressure) do not produce elevated ROS relative to low-Ψ_*f*_ control tasks, or if antioxidant supplementation (increasing β) does not modulate behavioral Ψ_*f*_ proxies, the ROS-friction coupling model is rejected.

### 3.4 Neural proxies

Neural proxies provide mechanistic depth but are not required for phase-1 falsification. They are treated as expansion modalities to test whether control-cost signatures generalize from low-cost measures to circuit-level dynamics.

#### 3.4.1 Perturbational complexity index (PCI)

PCI evidenced the complexity of the cortical response to transcranial magnetic stimulation perturbation, capturing the system's capacity for differentiated, integrated processing (Casali et al., 2013). We interpret PCI as indexing the residual control capacity of the selection/gating system: high PCI reflects high residual *P*_sel_, while low PCI reflects depleted capacity. PCI should decrease under high Ψ_*f*_ load and correlate negatively with behavioral Ψ_*f*_ proxies.

#### 3.4.2 Fisher information metric

The Fisher information matrix *g*_*F*_ captures the curvature of the neural state-space manifold, that is, how sharply the system's responses change as parameters vary. We propose that the empirical Fisher condition number log κ(*g*_*F*_), the Fisher volume term *logdet*(*g*_*F*_), and the maximum eigenvalue drift λ_max_(*g*_*F*_) serve as neural proxies for Ψ_*f*_ during state transitions (Amari, 2016). Specifically, transitions between cognitive states (e.g., task switches) should produce Fisher metric peaks *preceding* behavioral performance degradation, a “structural reconfiguration signal” that indexes friction before it manifests behaviorally.

#### 3.4.3 Theta-gamma coupling in the prefrontal cortex

Theta-phase/gamma-amplitude coupling (TGC) in prefrontal regions implements a multiplexing mechanism that constrains working memory capacity to approximately 5–7 items (Lisman and Idiart, 1995; Lundqvist et al., 2011). We interpret TGC integrity as an index of selection capacity: degraded TGC under high Ψ_*f*_ should predict working memory failures and executive breakdowns. Specifically, the phase-amplitude coupling modulation index (Tort et al., 2010) should decrease as behavioral Ψ_*f*_ composite scores increase.

#### 3.4.4 Thalamo–basal ganglia gating integrity

The thalamus–basal ganglia circuit functions as a selection gate that determines which candidate action trajectories are projected into overt execution. Gate integrity can be indexed through reinforcement-learning model parameters (learning rate, exploration-exploitation balance) derived from probabilistic reversal-learning tasks (Frank et al., 2004). Degraded gating (as in Parkinson's disease) should produce specifically elevated Ψ_*f*_ for action initiation while leaving cognitive Ψ_*f*_ relatively preserved.

### 3.5 Linguistic proxy: the modal mechanics probe

We introduce a novel, non-invasive proxy for Ψ_*f*_ based on the distribution of modal verbs in natural language production. We term this the *modal mechanics probe* (hypothesis H72 in the present framework).

#### 3.5.1 Theoretical rationale

Modal verbs encode speakers' orientation toward necessity, obligation, possibility, and desire (Palmer, 2001; van der Auwera and Plungian, 1998). The deontic–epistemic distinction in linguistic typology (Nuyts, 2001) maps directly onto an agency dimension: deontic modals (must, should, have to) express external or internalized obligations constraining action, while dynamic modals (can, want to, choose to) express the speaker's capacity or willingness to act. Developmental evidence shows that children acquire possibility modals before necessity modals, paralleling the emergence of agency and self-regulation (Papafragou, 2000), suggesting that modal usage tracks experienced control capacity from early in language development. We hypothesize that the balance between *constraint-oriented* modals (must, should, have to, cannot) and *possibility-oriented* modals (can, might, could, want to) reflects the speaker's experienced level of executive friction. High Ψ_*f*_ (experienced as constraint, obligation, and restriction) should bias language production toward constraint modals. Low Ψ_*f*_ (experienced as agency, possibility, and flow) should bias toward possibility modals.

This directionality is consistent with broader evidence that clinically relevant affective-control states leave measurable signatures in language production (Rude et al., 2004), including large-scale clinical NLP evidence that natural language markers can prospectively index depression risk (Eichstaedt et al., 2018). While these prior studies focused on pronoun use and general negative affect words rather than modal verbs specifically, the present proposal extends the same logic to a targeted, mechanistically interpretable subchannel: if control cost constrains action selection, and modal verbs encode the speaker's perceived action affordances, then modal distributions should function as a semantic readout of control-cost state. This bridge between psycholinguistic modal semantics and executive-control cost is explicitly exploratory and constitutes a novel cross-disciplinary hypothesis to be adjudicated by H72.

#### 3.5.2 Neurobiological plausibility

We do not treat μ_sem_ as a purely stylistic marker. Under cognitive-control and embodied language accounts, lexical selection under conflict depends on frontally mediated control allocation and policy constraints (Miller and Cohen, 2001; Diamond, 2013). On this view, constraint-heavy modal output is interpretable as a semantic spillover of high control demand, that is, a downstream linguistic footprint of constrained control budgets rather than a detached linguistic artifact.

#### 3.5.3 Operational definition

The semantic modal ratio μ_sem_ is defined as follows:

$$\begin{array}{l} \mu_{\text{s}\text{e}\text{m}}=\frac{\sum_{i}w_{i}\cdot \text{F}\text{r}\text{e}\text{q}\left({\text{c}\text{o}\text{n}\text{s}\text{t}\text{r}\text{a}\text{i}\text{n}\text{t}\;\text{m}\text{o}\text{d}\text{a}\text{l}}_{i}\right)}{\sum_{j}w_{j}\cdot \text{F}\text{r}\text{e}\text{q}\left({\text{p}\text{o}\text{s}\text{s}\text{i}\text{b}\text{i}\text{l}\text{i}\text{t}\text{y}\;\text{m}\text{o}\text{d}\text{a}\text{l}}_{j}\right)} \end{array}$$

where *w*_*i*_ *and w*_*j*_ are domain-specific weights (initially set to 1.0 for equal weighting), and Freq denotes frequency per 1,000 words in a transcribed speech sample. Constraint modals include: *must, have to, need to, should, ought to, cannot, must not*. Possibility modals include: *can, could, may, might, want to, would like to, choose to*.

#### 3.5.4 Reproducible scoring protocol and confound control

To make μ_sem_ scale-like rather than anecdotal, we use a residuali score:

$$\begin{array}{l} \log\mu_{\text{s}\text{e}\text{m}}=b_{0}+b_{1}\text{E}\text{d}\text{u}\text{c}\text{a}\text{t}\text{i}\text{o}\text{n}+b_{2}\text{P}\text{r}\text{o}\text{m}\text{p}\text{t}\text{T}\text{y}\text{p}\text{e}+b_{3}\text{I}\text{n}\text{t}\text{e}\text{r}\text{v}\text{i}\text{e}\text{w}\text{e}\text{r}\\ +b_{4}\text{A}\text{f}\text{f}\text{e}\text{c}\text{t}\text{L}\text{e}\text{x}\text{i}\text{c}\text{o}\text{n}\text{R}\text{a}\text{t}\text{i}\text{o}+b_{5}\text{T}\text{o}\text{k}\text{e}\text{n}\text{C}\text{o}\text{u}\text{n}\text{t}\\ +b_{6}\text{T}\text{y}\text{p}\text{e}\text{T}\text{o}\text{k}\text{e}\text{n}\text{R}\text{a}\text{t}\text{i}\text{o}+\varepsilon ,\\ \mu_{\text{s}\text{e}\text{m}}^{\text{a}\text{d}\text{j}}=\text{R}\text{e}\text{s}\text{i}\text{d}\text{u}\text{a}\text{l}\left(\log\mu_{\text{s}\text{e}\text{m}}\right) \end{array}$$ (10a)

This controls for major non-theoretical variance sources: education level, interview context, prompt wording, interviewer style, global affective-word prevalence, and lexical diversity. Recommended reliability checks are split-half agreement, inter-rater agreement for modal tagging (if manual annotation is used), and longitudinal intraclass correlation coefficient (ICC) in repeated sessions. To reduce floor/ceiling instability, analyses should exclude samples with fewer than 10 total modal tokens.

#### 3.5.5 Cross-language mapping protocol (Mandarin example)

Cross-linguistic adaptation should preserve *semantic role classes* (constraint vs. possibility), not literal word matching. For Mandarin Chinese, an initial inventory can be instantiated as follows:

- Constraint-oriented modals (Mandarin pinyin): bixu, dei, yao, yinggai, buneng, bude
- Possibility-oriented modals (Mandarin pinyin): keyi, neng, keneng, huoxu, xiang, yuanyi

Each language version should report (1) dictionary construction rules, (2) tokenization/segmentation pipeline, (3) ambiguity resolution rules (e.g., deontic vs. epistemic usage), and (4) measurement-invariance checks before pooling cross-language samples. The minimum pre-registered sequence is configural, then metric invariance (scalar optional); if metric invariance fails, confirmatory analyses remain language-specific and pooled estimates are treated as exploratory only.

#### 3.5.6 Methodological advantages

μ_sem_ is non-invasive, low-cost, and scalable to large corpora, including interview archives and longitudinal recordings. It is therefore suitable as a phase-1 marker in the core validation chain.

### 3.6 Convergent validity architecture

The central operationalization claim is that the four proxy classes converge on a single latent variable, Ψ_*f*_, rather than measuring four independent constructs. This claim is testable through structural equation modeling (SEM).

#### 3.6.1 Proposed model

The behavioral indicators draw on tasks that Miyake et al. (2000) showed load onto partially separable executive factors (shifting, inhibition, updating). We do not assume these sub-processes are identical; rather, we test whether a *second-order* Ψ_*f*_ factor accounts for shared variance across shifting (task-switch cost), inhibition (Stroop interference), and flexibility (WCST perseverative errors), with temporal discounting as an additional indicator from the effort-based decision literature. The confirmatory modeling sequence is therefore:

1. Within-behavioral CFA: fit a correlated three-factor Miyake-style model (shifting, inhibition, flexibility) and test whether a second-order general factor provides acceptable fit (comparative fit index, CFI, **>0.90**; root mean square error of approximation, RMSEA, **<0.08**).
2. Cross-modal single-factor CFA with four indicator classes:
  ° Behavioral second-order factor → Ψ_*f*_ (expected loading: λ > 0.5)
  ° Physiological composite (HRV recovery slope, inverse-coded) → Ψ_*f*_ (λ > 0.4)
  ° Neural composite (PCI, inverse-coded; Fisher condition number) → Ψ_*f*_ (λ > 0.4)
  ° Linguistic index (μ_sem_) → Ψ_*f*_ (λ > 0.3).

For staged validation, we use a nested modeling strategy. **Core model (phase 1/2):** behavioral second-order factor + linguistic + low-cost physiology. **Expansion model (phase 3):** add neural and ROS blocks and test whether they improve fit and prediction beyond the core model.

#### 3.6.2 Expected correlation structure

Within-domain correlations should be moderate to strong (*r* = 0.4-0.7); between-domain correlations should be weak to moderate (*r* = 0.20-0.45). We set these expectations conservatively relative to typical cross-modal correlations in the clinical psychophysiology literature. If between-domain correlations fall below *r* = 0.15 uniformly, the unitary latent variable interpretation is rejected in favor of independent constructs.

#### 3.6.3 Minimum survivable model if a single factor fails

The fallback is pre-registered rather than *post hoc*: (a) a two-factor model separating control-performance (behavioral + physiology) from semantic-constraint (linguistic, plus optional neural/expansion indicators), and (b) a bifactor model with one general factor plus modality-specific factors. The framework is retained only if the general factor shows stable loadings and predicts critical-load outcomes beyond modality factors; otherwise, the theory is downgraded to a family of modality-indexed frictions (${\text{Ψ}}_{f}^{\left(m\right)}$) rather than one unitary latent s.

#### 3.6.4 Discriminant validity

**Figure 2:**
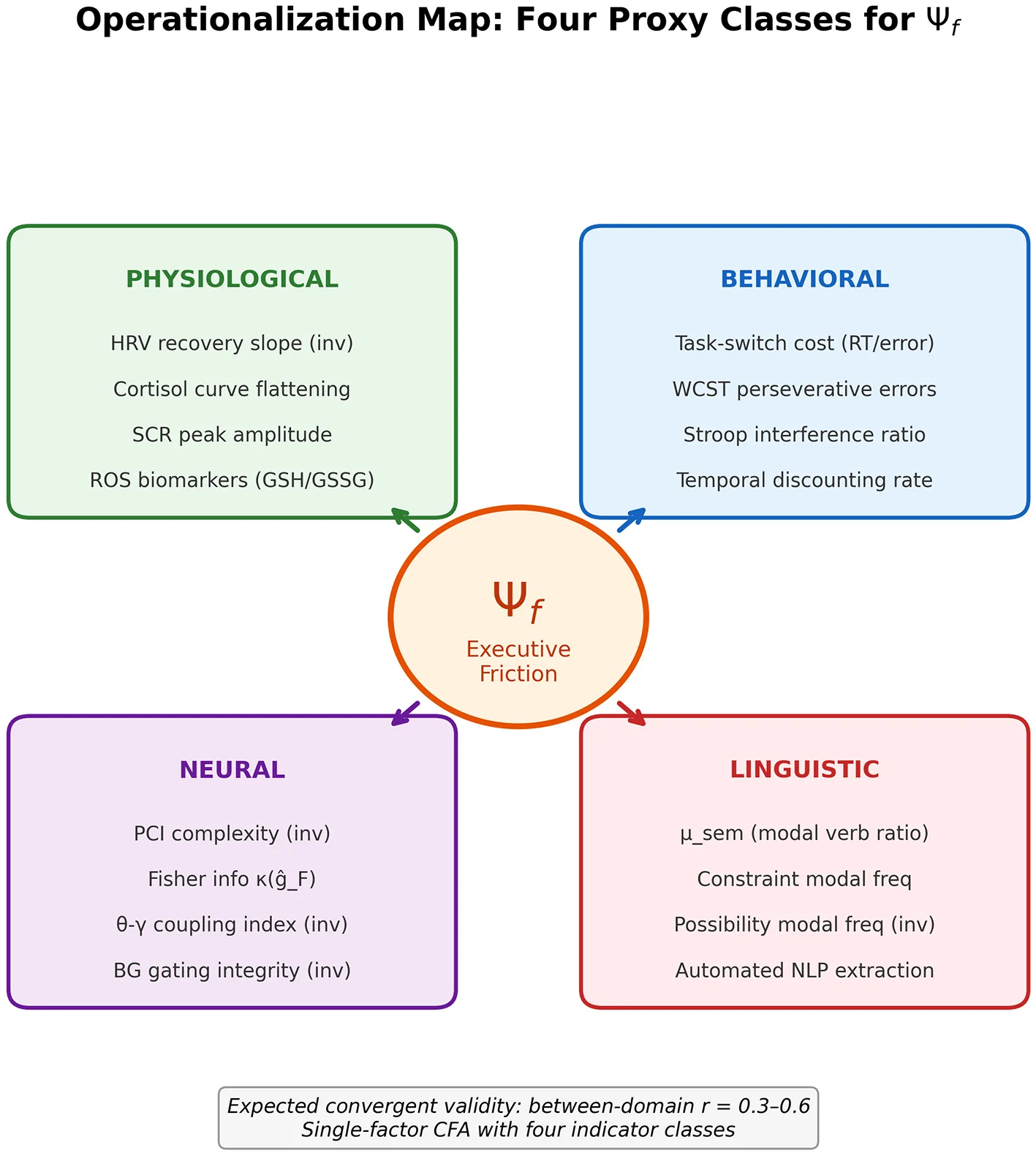
*Cross-modal operationalization map for executive friction (*Ψ_*f*_*)*. Central latent construct with four proxy classes. Core validation chain: behavioral, linguistic, and low-cost physiology (HRV/SCR/cortisol). Expansion chain: ROS biomarkers and neural proxies (PCI, Fisher information, theta-gamma coupling, basal ganglia gating). Arrows indicate predicted direction of association; “(inv)” denotes inverse coding.

Ψ_*f*_ should be empirically distinguishable from general cognitive ability (IQ), general psychopathology (*p*-factor), and trait neuroticism. We predict that Ψ_*f*_ shows incremental validity over these constructs in predicting task-switching cost and clinical executive dysfunction (Figure 2).

## 4 Clinical mapping: across disorders Ψ_f_

The Ψ_*f*_ framework generates disorder-specific predictions that go beyond the generic claim that “executive function is impaired.” Each clinical condition is modeled as a distinct perturbation of the Ψ_*f*_ dynamics, producing a characteristic *friction signature* across the four proxy classes.

### 4.1 Major depressive disorder: sustained friction with oxidative coupling

In the Ψ_*f*_ framework, major depressive disorder (MDD) is modeled as a state of *chronic friction elevation* in which *dΨ*_*f*_/*dt* remains positive over extended periods, leading to progressive exhaustion of selection power and the accumulation of oxidative stress.

#### 4.1.1 Mechanism

The depressed agent's learned model may be accurate or even hyperaccurate (“depressive realism”; Alloy and Abramson, 1979): the agent correctly identifies what actions would be beneficial. However, the friction cost of *initiating* state transitions (${\text{Ψ}}_{f}^{\text{a}\text{c}\text{t}\text{i}\text{o}\text{n}}$) chronically exceeds the available residual selection power ($P_{\text{s}\text{e}\text{l}}^{residual}$). The agent enters a stable low-energy attractor in which inaction minimizes acute distress (*dΨ*_*f*_/*dt* ≈ 0) at the cost of chronic friction accumulation.

The ROS coupling equation (Equation 9) generates a specific pathophysiological chain: sustained Ψ_*f*_ → elevated metabolic demand → increased mitochondrial ROS production → oxidative damage → inflammatory signaling → further reduction of *P*_sel_ through immune-mediated neuromodulatory changes. This positive feedback loop is consistent with the inflammatory hypothesis of depression (Maes et al., 2011; Miller and Raison, 2016) and provides a mechanistic link between the cognitive and immunological dimensions of MDD. In this study, however, these arrows are treated as **directional hypotheses subject to prospective testing rather than established causal pathways**; cross-sectional data can establish compatibility but not causal direction.

#### 4.1.2 Predicted friction signature

**Figure 3:**
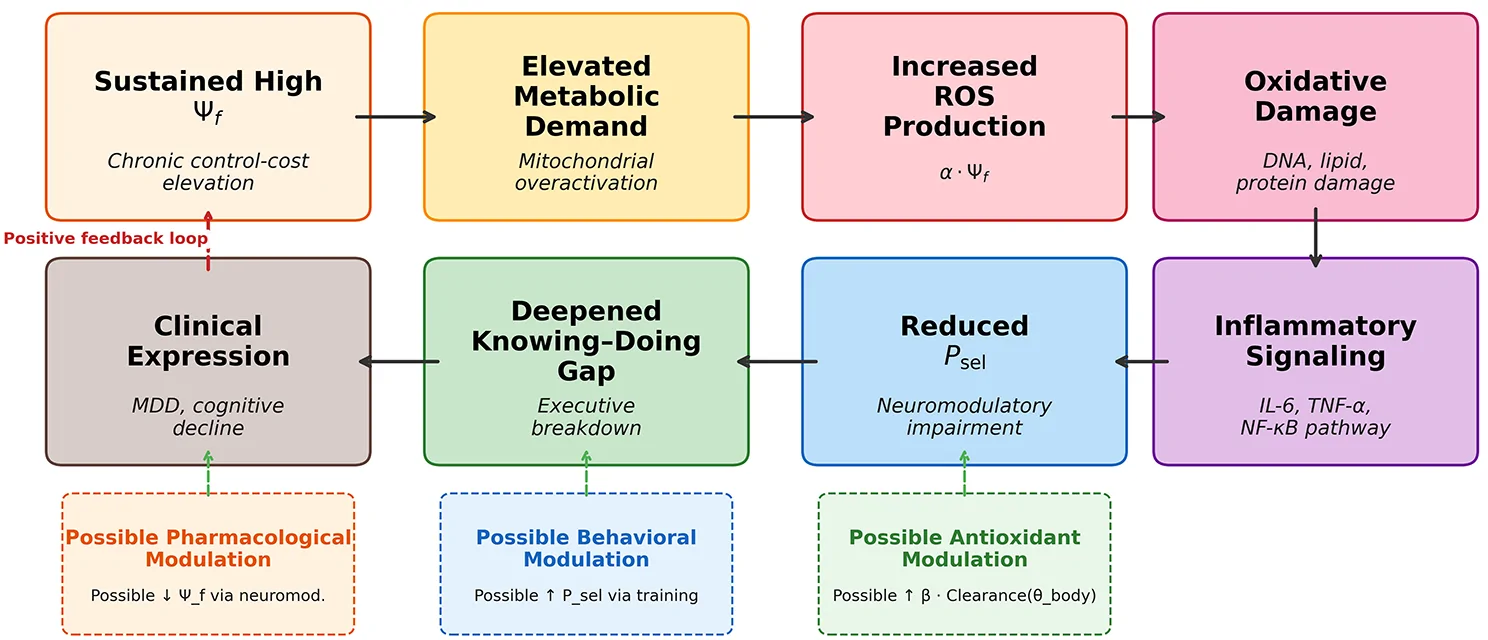
*Proposed oxidative feedback loop linking sustained executive friction to clinical features*. Schematic diagram showing a proposed loop from sustained high Ψ_*f*_ through elevated metabolic demand, increased ROS production, oxidative damage, inflammatory signaling, reduced selection power (*P*_sel_), and a deepened knowing-doing gap (dashed red feedback arrow). Dashed boxes at bottom indicate three possible modulation points: pharmacological (↓ Ψ_*f*_), behavioral (↑ *P*_sel_), and antioxidant (↑ clearance).

Core chain prediction for depression is: (a) elevated behavioral Ψ_*f*_ composite, particularly temporal discounting and task-switching cost; (b) reduced HRV recovery slope and altered SCR/cortisol profile; and (c) elevated μ_sem_ with predominance of obligation/constraint modals. Expansion prediction is reduced PCI/degraded theta-gamma coupling plus elevated ROS biomarkers (reduced GSH/GSSG ratio, elevated MDA) (Figure 3).

### 4.2 Obsessive-compulsive disorder: switching viscosity

OCD is modeled as a pathological elevation of *switching viscosity* η, the resistance of the parameter space to state transitions. This is not a new parameter but the same viscosity introduced behaviorally in Section 3.1, where perseverative errors were said to index η ∝ ∂Ψ_*f*_/∂θ: the sensitivity of friction to changes in the control parameters. High viscosity means that even slight changes in the parameters incur a large increase in friction, so the system resists transitions. The compulsive behavior occupies a local minimum in the friction landscape with extremely steep walls, making it energetically prohibitive to transition to alternative states.

#### 4.2.1 Mechanism

We model OCD switching viscosity using a network-modulated gradient flow:

$$\begin{array}{l} \frac{d\theta_{i}}{dt}=-\frac{\nabla_{i}\Phi \left(\theta \right)}{1+\lambda d_{i}}+\epsilon_{i}\left(t\right),\;\lambda 0>0\; \end{array}$$

where Φ(θ) is the friction potential, *d*_*i*_ is the network degree (or weighted centrality) of node *i*, and ϵ_*i*_(*t*) is noise. High-centrality nodes update more slowly because the effective step size is reduced by ${\left(1+\lambda d_{i}\right)}^{-1}$. Local trapping strength is evidenced by curvature as expressed in the following:

$$\begin{array}{l} {\text{Ψ}}_{f}^{\text{s}\text{w}\text{i}\text{t}\text{c}\text{h}}\left(\theta_{i}\right)\propto \lambda_{\max}\left(\nabla_{\theta_{i}}^{2}\Phi \left(\theta \right)\right) \end{array}$$

so compulsive states correspond to deep, high-curvature local wells that raise transition cost.

#### 4.2.2 Predicted friction signature

OCD is characterized by: (a) dramatically elevated task-switching cost *specifically* for uncertainty- and contamination-related stimuli; (b) elevated SCR during obsession-related cues (acute *dΨ*_*f*_/*dt* spikes); and (c) the highest μ_sem_ values, dominated by “must/have to” constructions. Expansion prediction is relatively preserved PCI (system not globally bandwidth-depleted but dynamically stuck). The proposed discriminator for depression is elevated *switching* friction with comparatively preserved maintenance capacity. We stress that this is a claim about relative profile, not a clean dividing line: depression also impairs task-switching, so the two conditions overlap on any single measure of switching. The framework's differentiating bet is therefore on the *pattern* across proxies (in OCD, cue-specific switching cost and the highest constraint-modal μ_sem_ against comparatively preserved maintenance and global PCI; in MDD, broader maintenance-side involvement with cortisol flattening and oxidative coupling) rather than on switching cost alone. Because this overlap poses a genuine boundary risk for the account, we return to it explicitly in Section 6.3.

### 4.3 Parkinson's disease: selection gate degradation

Parkinson's disease (PD) is modeled as a degradation of the thalamo-basal ganglia selection gate, the circuit that converts motor plans stored in slower control variables into initiated actions in the active motor state.

#### 4.3.1 Mechanism

The dopaminergic signal-to-noise ratio in the striatum serves as a gain parameter for the gate. As dopaminergic neurons in the substantia nigra degenerate, the gate's capacity to distinguish candidate actions diminishes, and initiation friction rises (Frank et al., 2004; Redgrave et al., 2010). We use a dimensionless embodied anchoring index:

$$\begin{array}{l} {\tilde{\kappa }}_{\text{b}\text{o}\text{d}\text{y}}\left(t\right)=\frac{\text{G}\text{r}\text{i}\text{p}\text{F}\text{o}\text{r}\text{c}\text{e}\left(t\right)/F_{0}}{{\text{Ψ}}_{f}^{\text{i}\text{n}\text{i}\text{t}\text{i}\text{a}\text{t}\text{i}\text{o}\text{n}}\left(t\right)/{\text{Ψ}}_{0}} \end{array}$$

where *F*_0_ and Ψ_0_ are participant-specific normalization constants (e.g., baseline grip force and baseline initiation friction estimate). The anchoring index is not an independent construct: its denominator, ${\text{Ψ}}_{f}^{\text{i}\text{n}\text{i}\text{t}\text{i}\text{a}\text{t}\text{i}\text{o}\text{n}}$, is simply the action-initiation friction ${\text{Ψ}}_{f}^{\text{a}\text{c}\text{t}\text{i}\text{o}\text{n}}$ from the knowing–doing inequality (Equation 7) applied to the motor domain, and the ratio expresses how much realized bodily output (grip force) a unit of initiation friction actually yields. As dopaminergic availability decreases, initiation friction rises, and ${\tilde{\kappa }}_{\text{b}\text{o}\text{d}\text{y}}\to 0$: the agent can generate a motor plan but fails to anchor execution in embodied output. This is the motor-domain instance of the general knowing–doing gap, where “knowing” is the intact motor plan and “can't do” is the failure to pay ${\text{Ψ}}_{f}^{\text{a}\text{c}\text{t}\text{i}\text{o}\text{n}}$.

#### 4.3.2 Predicted friction signature

PD is characterized by: (a) elevated task-switching cost for motor tasks with relatively preserved cognitive switching (early stages); (b) elevated SCR during motor initiation attempts with less pronounced endocrine flattening; and (c) μ_sem_ elevation concentrated in motor-action expressions. Expansion prediction is region-specific PCI reduction in the motor cortex with relative prefrontal preservation.

For empirical PD cohorts, a minimal adjustment set should include dopaminergic medication state (ON/OFF and levodopa-equivalent daily dose), disease duration, motor severity (e.g., UPDRS), sleep status, and major psychiatric comorbidity.

### 4.4 Differential prediction table

The clinical utility of Ψ_*f*_ depends on its capacity to generate *disorder-specific* predictions, not merely generic “executive dysfunction” claims. Table 2 summarizes the predicted friction signatures across conditions.

**Table 2:** Predicted Ψ_*f*_ proxy signatures across clinical conditions.

Proxy	HC (reference)	MDD (predicted deviation)	OCD (predicted deviation)	PD (predicted deviation)	If wrong, implication
Task-switch cost (RT)	reference	moderate increase	**marked increase (domain-specific)^†^**	**marked motor increase; limited cognitive increase in early PD^†^**	If PD shows generalized (not motor-specific) elevation, gate-degradation model needs revision
Perseverative errors	reference	mild-to-moderate increase	marked increase	small increase	—
Temporal discounting	reference	moderate increase	small-to-moderate increase	little or no systematic increase	—
HRV recovery slope	reference	slower recovery	mildly slower recovery	mildly slower recovery	—
Cortisol curve	reference	flatter diurnal profile	near-control range	near-control range	—
SCR during conflict	reference	often blunted or dysregulated	elevated for obsession-relevant cues	elevated during initiation attempts	—
ROS biomarkers (expansion)	reference	elevated in expansion studies	near-control range	mild elevation possible	—
PCI (expansion)	reference	reduced in expansion studies	**near-control range^†^**	motor-circuit reduction more likely than global reduction	If OCD shows globally reduced PCI, “stuck but not depleted” model fails
Theta-gamma coupling (expansion)	reference	degraded in expansion studies	near-control range	relatively preserved cognitive coupling	—
μ_sem_	reference	moderate elevation	**highest constraint-dominant elevation^†^**	elevation concentrated in action-initiation descriptions	If OCD does not show highest μ_sem_, switching-viscosity linguistic prediction fails

Bold entries marked with ^†^ denote the primary disorder-discriminating predictions used in the paired dissociation tests.

Core-chain inference in this study is based primarily on behavioral + linguistic + low-cost physiological rows. ROS and neural rows are treated as expansion targets for later-phase adjudication.

To sharpen falsifiability, we propose two discriminator patterns. **Pattern A (OCD dissociation):** OCD should show a clearer constraint-dominant μ_sem_ profile than controls and typically than MDD, while PCI remains near the control range in expansion cohorts. **Pattern B (PD motor selectivity):** PD should show substantially larger motor-switch impairment than cognitive-switch impairment in early-stage cohorts. In contrast, single-resource formulations (including standard single-task expected value of control (EVC) fits or undifferentiated global-deficit accounts) typically predict more monotonic cross-domain impairment and weaker dissociation in these paired contrasts.

**Figure 4:**
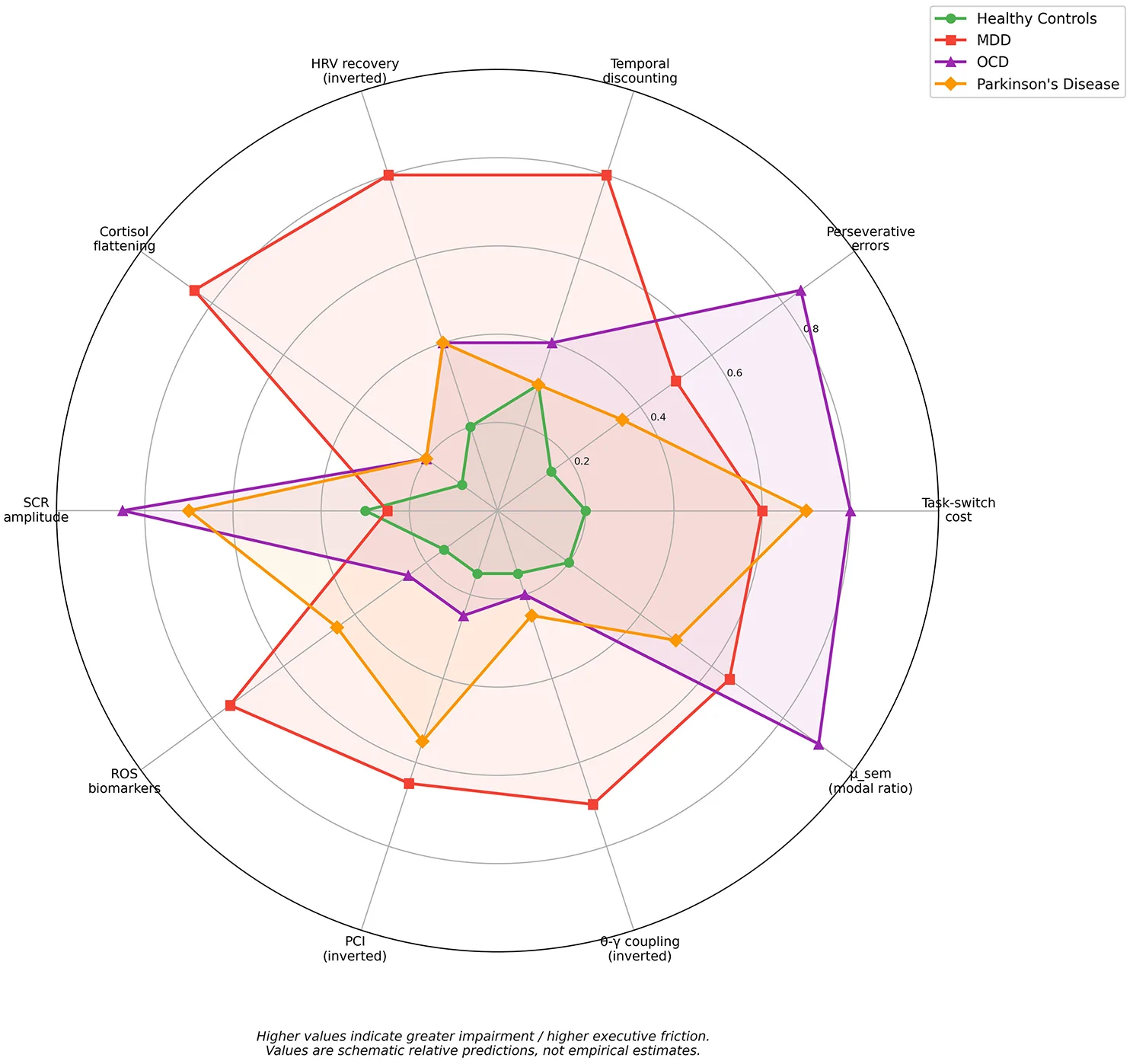
*Schematic predicted* Ψ_*f*_ *proxy signatures across clinical conditions*. Radar chart showing schematic relative profiles for healthy controls (green), major depressive disorder (red), obsessive-compulsive disorder (purple), and Parkinson's disease (orange) across ten proxy measures. Higher values indicate greater impairment or higher executive friction. Values are schematic relative predictions, not empirical estimates.

These predictions are falsifiable: if, for example, OCD patients do not show the highest μ_sem_ values, or if PD patients show generalized rather than motor-specific switching cost elevation, the disorder-specific Ψ_*f*_ models require revision (Figure 4).

### 4.5 Competing explanations and decisive phase-2 tests

To reduce over-interpretation risk, each disorder signature is paired with a concrete competing explanation and a decisive phase-2 contrast:

- **MDD** vs. generic symptom-severity accounts. Decisive test: whether high Ψ_*f*_ profile predicts a specific combination (high temporal discounting + slow HRV recovery + elevated μ_sem_) rather than uniform global impairment alone.
- **OCD** vs. generalized anxiety/rumination accounts. Decisive test: domain-specific switching viscosity plus highest μ_sem_ under preserved global PCI-range indices in expansion cohorts.
- **PD** vs. global psychomotor slowing. Decisive test: motor-selective switch impairment with comparatively preserved cognitive switching in early-stage cohorts, plus initiation-specific autonomic spikes.

If these contrasts fail, the clinical mapping should be downgraded to transdiagnostic severity effects rather than disorder-specific signatures.

## 5 Falsifiable hypotheses and proposed experimental protocols

We now present five preregistration-ready hypotheses derived from the Ψ_*f*_ framework. Each hypothesis specifies the prediction, the falsification criterion, the required sample size, and the core measurement protocol. We emphasize that these hypotheses are designed to be *individually falsifiable*: rejection of any single hypothesis does not invalidate the entire framework but constrains its scope.

### 5.1 Hypothesis H72: modal verb patterns reflect executive friction

#### 5.1.1 Prediction

Within-subject adjusted modal ratio $\mu_{\text{s}\text{e}\text{m}}^{\text{a}\text{d}\text{j}}$ (Equation 10a) is higher in **self-decision narratives** than in **neutral factual retell** prompts, and the context contrast $\Delta \mu_{\text{s}\text{e}\text{m}}^{\text{a}\text{d}\text{j}}$ is expected to show a small-to-moderate positive association with the behavioral Ψ_*f*_ composite (task-switch cost + Stroop interference + perseverative errors) and inverse association with HRV recovery slope. We further predict incremental validity after adding symptom controls (PHQ-9, GAD-7, or equivalent), affective-word ratio, and block-wise appraisal ratings.

#### 5.1.2 Protocol

*N* = 120 healthy adults, tested in three sessions over 6 weeks. Each session includes: (1) two fixed prompt blocks (neutral factual retell and self-decision narrative; total 15 minutes), recorded and transcribed; (2) behavioral battery (task switching, Stroop, WCST); and (3) HRV + SCR recording (cortisol optional for the core chain). Prompt administration is standardized: factual block uses a fixed non-evaluative script (e.g., “describe yesterday's routine chronologically”), decision block uses a fixed self-choice script (e.g., “describe an unresolved decision and candidate actions”), each with fixed speaking duration and counterbalanced order across sessions. After each block, participants provide a 0–100 challenge/threat appraisal score used in Equation 4-linked analyses. μ_sem_ is extracted using a pre-registered modal dictionary and residual according to Equation 10a. Interviewer identity is modeled as a random effect (a random intercept plus a context slope when estimable). Samples with fewer than 10 modal tokens are excluded *a priori*.

#### 5.1.3 Statistical analysis

Multilevel models with random intercepts and slopes are used for within-subject association tests. Primary fixed effects are context (decision vs. factual), $\Delta \mu_{\text{s}\text{e}\text{m}}^{\text{a}\text{d}\text{j}}$, block-wise appraisal, and their links to behavioral/HRV outcomes. Incremental validity is tested via Δ*R*^2^ after adding PHQ-9 and GAD-7 (Kroenke et al., 2001; Spitzer et al., 2006) or equivalent symptom scales, plus affective-word ratio. If multilingual cohorts are used, configural/metric invariance is tested before pooled confirmatory inference. Test-retest reliability is assessed via ICC for both raw μ_sem_ and adjusted $\mu_{\text{s}\text{e}\text{m}}^{\text{a}\text{d}\text{j}}$.

#### 5.1.4 Falsification criterion

We specify a primary and a secondary test. The *primary* test is construct validity: whether a decision vs. factual context effect is observed for $\mu_{\text{s}\text{e}\text{m}}^{\text{a}\text{d}\text{j}}$, which is the within-subject signature the framework predicts. The *secondary* test is incremental utility: whether adjusted $\mu_{\text{s}\text{e}\text{m}}^{\text{a}\text{d}\text{j}}$ adds meaningful predictive variance beyond symptom and affect controls. Full support for the linguistic probe requires both. If *either* test fails, the probe is downgraded and retained only as an exploratory, secondary indicator rather than a core-chain marker. If *both* tests fail, μ_sem_ is removed from the core model entirely. This graded rule ties the primary decision to construct validity while still crediting incremental utility as secondary evidence.

#### 5.1.5 Planning note

The initial planning value is *N* = 120 across three time points, sized for stable estimation of small-to-moderate within-subject associations under plausible repeated-measures reliability assumptions. Sample sizes were informed by effect-size benchmarks from the task-switching, executive-function, and clinical psychophysiology literatures, while exact power tolerances are intended to be fixed in the public preregistration and accompanying scripts before data collection.

### 5.2 Hypothesis H-NEURO-EXEC-01: non-linear executive cost under high Ψ_f_

#### 5.2.1 Prediction

Task-switching cost increases non-linearly as a function of concurrent normalized load. The confirmatory estimator is ρ^*^ (Equation 7c), with ratio-based ρ (Equation 7a) as a sensitivity analysis. Specifically, a changepoint model (Equation 7b) should show a meaningfully better fit than a linear model and yield an identifiable critical point $\rho_{c}^{*}$ (and concordant ρ_*c*_ in sensitivity checks).

#### 5.2.2 Protocol

*N* = 80 healthy adults in a within-subject design. Ψ_*f*_ load is manipulated at four levels: (1) single-task switching, (2) low concurrent load, (3) high concurrent load, and (4) social-evaluative stress. Task-switching cost is measured at each level; HRV and SCR are recorded to estimate concurrent load. For phase-1 estimation, baseline friction is operationalized as a latent composite from $\mu_{\text{s}\text{e}\text{m}}^{\text{a}\text{d}\text{j}}$, resting HRV (inverse-coded), and baseline switch cost; *P*_sel_ is proxied by normalized HRV/SCR recovery. Primary confirmatory analyses use ρ^*^ (Equation 7c); ratio-based ρ (Equation 7a) is pre-specified sensitivity analysis.

#### 5.2.3 Falsification criterion

If a linear model provides a comparable or better fit in the **primary** ρ^*^analysis, or if no positive $\rho_{c}^{*}$ is identifiable, the bandwidth-saturation account is rejected in favor of additive resource models. Ratio-based ρ results are reported as sensitivity checks.

### 5.3 Hypothesis H-MET-01: free choice metabolic premium

#### 5.3.1 Prediction

Decisions that require overriding default or habitual responses (operationalized as choosing the counter-habitual option in a trained stimulus-response compatibility task) produce higher *autonomic control cost* (primary endpoint: SCR amplitude) than decisions aligned with trained responses, and this cost differential is predicted by the individual's behavioral Ψ_*f*_ composite score. Blood glucose change is treated as a secondary exploratory endpoint because the glucose-specific ego-depletion mechanism remains contested (Baumeister et al., 1998; Hagger et al., 2016; Vadillo et al., 2016).

#### 5.3.2 Protocol

*N* = 60 healthy adults. Phase 1 (training): participants learn a stimulus-response mapping over 200 trials. Phase 2 (test): on 50% of trials, participants are instructed to execute the *opposite* of their trained response. SCR is recorded continuously as the primary physiological endpoint; capillary blood glucose (pre/post Phase 2) is collected as a secondary exploratory endpoint. Behavioral Ψ_*f*_ composite is measured in a separate session.

#### 5.3.3 Falsification criterion

If SCR amplitude does not differ between habitual and counter-habitual responses, or if the SCR difference shows no reliable relation to the Ψ_*f*_ composite, the primary metabolic-premium hypothesis is rejected. A null glucose effect alone does not falsify H-MET-01 but constrains substrate-level interpretations.

### 5.4 Hypothesis H-CLIN-DEP-01: ROS– coupling in depression Ψ_f_

#### 5.4.1 Prediction

In a sample of MDD patients (*n* = 40) and matched healthy controls (*n* = 40), the behavioral Ψ_*f*_ composite score predicts ROS biomarker levels (GSH/GSSG ratio) with a positive association of at least small-to-moderate magnitude in the MDD group. HRV mediation is tested as a compatibility model in cross-sectional data and is not interpreted as causal directionality.

#### 5.4.2 Protocol

Cross-sectional comparison. Each participant completes the full behavioral task battery (§3.1), provides salivary cortisol and blood samples for ROS assays, and undergoes HRV recording. MDD diagnosis is confirmed via the Structured Clinical Interview for DSM-5 (SCID-5). Predefined exclusion/covariate set includes acute inflammatory disease, systemic steroid or high-dose antioxidant use, unstable medical illness, substance dependence, antidepressant class/dose (e.g., selective serotonin reuptake inhibitors, SSRIs, or serotonin–norepinephrine reuptake inhibitors, SNRIs), illness duration, episode severity, anxiety comorbidity, and sleep-quality index.

#### 5.4.3 Falsification criterion

If the Ψ_*f*_ composite–GSH/GSSG association is negligible in the MDD group, or if the HRV compatibility model contributes no interpretable structure, the ROS-friction coupling model is rejected for depression.

### 5.5 Hypothesis H-CLIN-OCD-01: maximal in OCD μ_sem_

#### 5.5.1 Prediction

In a three-group comparison (OCD, *n* = 30; MDD, *n* = 30; healthy controls, *n* = 30), we predict $\mu_{\text{s}\text{e}\text{m}}^{\text{O}\text{C}\text{D}}>\mu_{\text{s}\text{e}\text{m}}^{\text{M}\text{D}\text{D}}>\mu_{\text{s}\text{e}\text{m}}^{\text{c}\text{o}\text{n}\text{t}\text{r}\text{o}\text{l}\text{s}}$, driven specifically by elevated frequency of constraint modals. These two inequalities are not equivalent, and it is worth stating what each one buys. The contrast **OCD** **>** **controls** would establish only the weaker, more general claim that friction leaves a constraint-modal signature in language at all; both OCD and MDD are elevated-friction states, so an elevation over healthy controls is expected under the framework but does not by itself distinguish disorders. The contrast **OCD** **>** **MDD** is the stronger and genuinely disorder-differentiating claim: it is what the switching-viscosity account specifically predicts (constraint-dominant modal output should peak where switching is most rigidly trapped), and it is the result that would separate Ψ_*f*_ from a generic “any distress raises constraint language” account.

#### 5.5.2 Protocol

Each participant completes a 20-minute semi-structured clinical interview (using the Yale-Brown Obsessive-Compulsive Scale for the OCD group, Hamilton Depression Rating Scale for the MDD group) plus a standardized free-narrative prompt (“Describe your daily routine and the challenges you face”). Transcripts are analyzed for μ_sem_. Minimal adjustment set includes SSRI/antipsychotic augmentation status, benzodiazepine use, illness duration, anxiety and sleep comorbidity indices, and interviewer random effects.

#### 5.5.3 Falsification criterion

The two claims are rejected at different levels. If OCD μ_sem_ does not reliably exceed *healthy control* μ_sem_, the linguistic proxy fails even its weak form, and the modal signature is abandoned as a friction marker. If OCD exceeds controls but does *not* reliably exceed *MDD*, the weak form survives, but the disorder-differentiating claim is rejected: μ_sem_ is then retained as a transdiagnostic friction index rather than as an OCD-specific signature. Only the joint result OCD > MDD > controls supports the full switching-viscosity prediction.

### 5.6 Proposed validation roadmap

We propose a three-phase validation program:

#### 5.6.1 Phase 1 (core low-cost mechanism test)

Execute H72 and H-NEURO-EXEC-01 using behavioral tasks, $\mu_{\text{s}\text{e}\text{m}}^{\text{a}\text{d}\text{j}}$, HRV, and SCR. Objective: identify whether a stable latent Ψ_*f*_ factor and a measurable critical point $\rho_{c}^{*}$ exists in healthy cohorts (with ratio-based ρ_*c*_ as a sensitivity check). *N*_*total*_ ≈ 200. Estimated timeline: 12 months.

#### 5.6.2 Phase 2 (core clinical generalization)

Execute H-CLIN-OCD-01 and H-MET-01 with the same low-cost core chain, extending to clinical and quasi-clinical groups while preserving protocol simplicity. *N*_*total*_ ≈ 150. Estimated timeline: 18 months.

#### 5.6.3 Phase 3 (high-cost optional adjudication)

Execute H-CLIN-DEP-01 with ROS assays and neural expansion markers (e.g., PCI, theta-gamma coupling, Fisher-geometry metrics) to test whether costly modalities add explanatory value beyond the core chain. These modalities are explicitly optional adjudicators, not prerequisites for validating the core model. *N*_*total*_ ≈ 200. Estimated timeline: 24 months.

At preregistration release, the public repository will include: modal dictionaries by language, preprocessing/tokenization scripts, model-specification files (core and fallback), and simulation scripts used to justify decision thresholds (Table 3).

**Table 3:** Falsification criteria summary.

Hypothesis	Tier	Core prediction	Falsification criterion	Required N	Key measures
H72	Core	Context-sensitive $\mu_{\text{s}\text{e}\text{m}}^{\text{a}\text{d}\text{j}}$ (primary) + incremental validity (secondary)	Either test fails → downgrade to exploratory; both fail (no context effect and no meaningful incremental variance over symptom/affect controls) → remove from core	120	Adjusted modal ratio, RT, HRV/SCR, symptom scales
H-NEURO-EXEC-01	Core	Nonlinear cost increase with identifiable $\rho_{c}^{*}$ (primary)	Linear fit dominates or no $\rho_{c}^{*}$	80	Task-switch × load, HRV/SCR, baseline latent score
H-MET-01	Core-extension bridge	Counter-habitual choice increases SCR (glucose exploratory)	No SCR difference or no reliable SCR-Ψ_*f*_ link	60	SCR (primary), glucose (secondary), compatibility task
H-CLIN-OCD-01	Core clinical	Highest μ_sem_ in OCD	No reliable OCD elevation on μ_sem_	90 (three groups of 30 participants)	Modal ratio, clinical interview
H-CLIN-DEP-01	Expansion	ROS predicted by Ψ_*f*_ in MDD	Negligible Ψ_*f*_-ROS association	80 (40+40)	GSH/GSSG, task battery, HRV

Primary-endpoint decision rules: directional primary tests are one-sided (α = 0.025); secondary tests use Benjamini-Hochberg FDR control (*q* < 0.05). Predefined primary endpoints are: H72 (context effect and incremental validity), H-NEURO-EXEC-01 ($\rho_{c}^{*}$ identification), H-MET-01 (SCR contrast), H-CLIN-OCD-01 (group contrast on μ_sem_), and H-CLIN-DEP-01 (Ψ_*f*_-ROS association). Ratio-based ρ inference is explicitly sensitivity-only.

**Figure 5:**
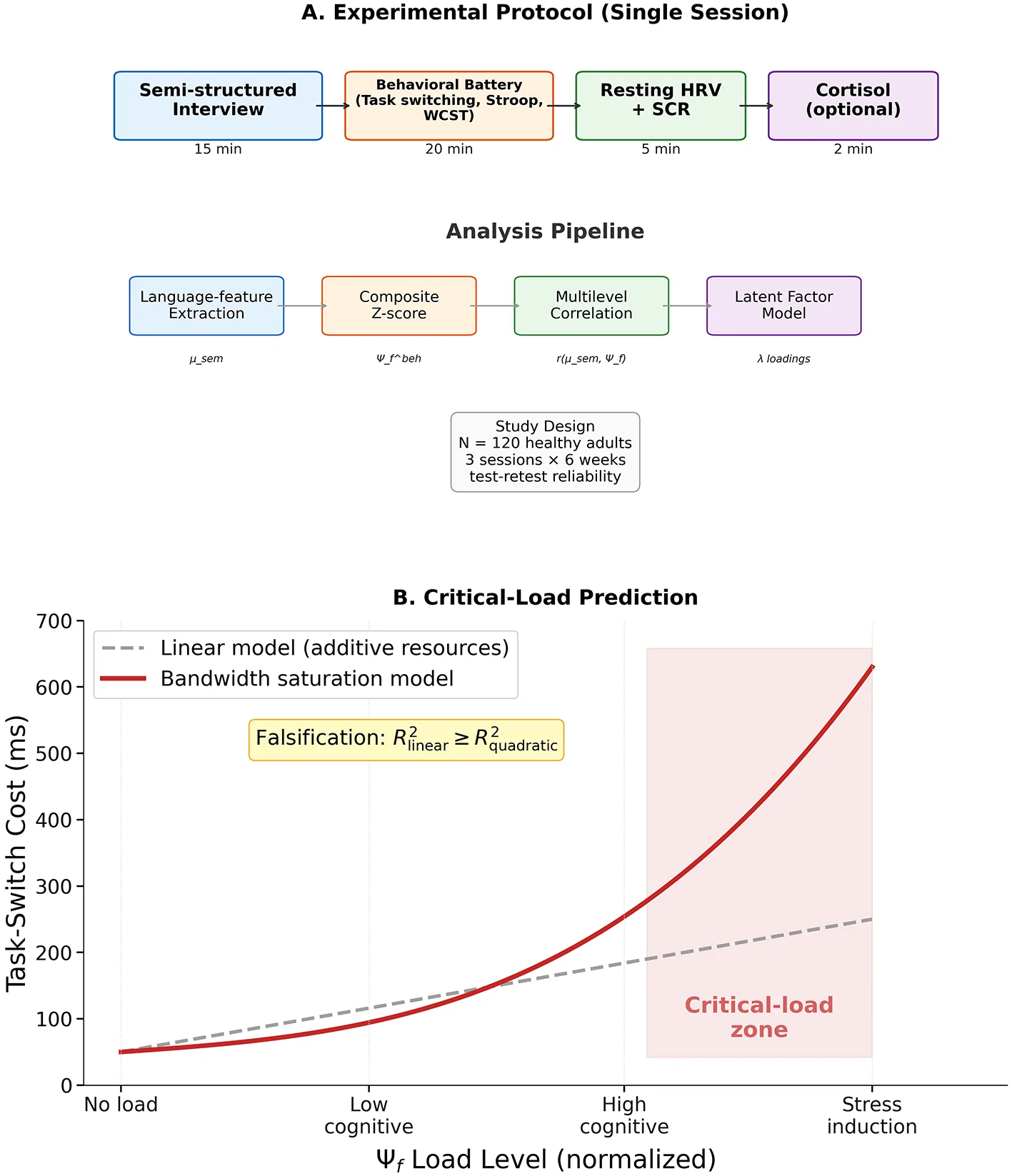
*Core-chain protocol and critical-load prediction*. **(A)** Low-cost protocol for H72 and H-NEURO-EXEC-01, integrating two prompt contexts (neutral factual retell vs self-decision narrative), behavioral battery, HRV/SCR recording, appraisal ratings, and NLP extraction for latent-factor estimation. Baseline load is estimated as $\rho^{*}=z\left({\text{Ψ}}_{f}^{baseline,\;latent}\right)-z\left(P_{\text{s}\text{e}\text{l}}^{proxy}\right)$ (Equation 7c) in primary analyses; ratio-based ρ is sensitivity-only. **(B)** Predicted task-switch cost as a function of normalized load ρ: the saturation model (solid red) includes a critical point ρ_*c*_ and nonlinear escalation into a “know but can't do” zone, while the additive model (dashed gray) predicts linear increase.

The one-sided choice is justified by preregistered directional monotonic predictions implied by Equations 4, 5, 7; all primary effects are also reported with two-sided sensitivity analyses. Exact small-effect cutoffs, model-selection tolerances, and simulation-based design anchors are intended to be fixed in the public preregistration and accompanying scripts before data collection, rather than treated as universal constants in the present theory article (Figure 5).

## 6 Discussion

### 6.1 Theoretical implications

The Ψ_*f*_ framework addresses a persistent gap in cognitive neuroscience: the absence of a cross-modal cost quantity that unifies behavioral, physiological, neural, and linguistic signatures of executive breakdown under a formal selection-budget constraint. By deriving the knowing–doing gap as bandwidth saturation (Equation 7) rather than as a domain-specific failure, the framework generates testable predictions that current models (cognitive load theory, FEP, IIT, allostatic load) do not individually produce.

The key theoretical advance is the *decoupling of knowledge from capacity*. In existing frameworks, executive failure is typically attributed to degraded representations (the agent doesn't know), degraded attention (the agent can't focus), or degraded motivation (the agent doesn't want to). The Ψ_*f*_ framework introduces a fourth possibility: the agent knows, can focus, and wants to act, but the *cost of converting knowledge into action* exceeds available resources. This bandwidth saturation account explains why patients often report the phenomenology of “knowing exactly what to do but being unable to do it,” a subjective experience that is puzzling under representational, attentional, or motivational accounts.

### 6.2 Comparison with existing frameworks

Table 4 compares Ψ_*f*_ with five existing frameworks across seven evaluation criteria.

**Table 4:** Framework comparison.

Feature	Cognitive load theory	Allostatic load	Free energy principle	EVC	IIT (Φ)	Present framework (Ψ_f_)
Cross-modal operationalization	No (behavioral only)	Partial (physiological)	Partial (neural + computational)	Partial (behavioral + computational; current formulations)	No (neural only)	Yes (4 proxy classes)
Formal equations	Minimal	No	Yes	Yes	Yes	Yes
Dynamic cost function	No (static)	Yes (cumulative)	Yes (instantaneous)	Yes (cost-benefit allocation)	No (snapshot)	Yes (accumulated + derivative)
Clinical disorder specificity	No	Partial	Partial	Partial (task-level, non-nosological)	No	Yes (Table 2)
Linguistic probe	No	No	No	No	No	Yes (μ_sem_)
Bandwidth saturation model	No	No	Implicit	Not explicit	No	Yes (Equation 7)
Explains knowing–doing gap	Partially	No	Partially	Partially	No	Yes

This comparison is not intended to argue that Ψ_*f*_ replaces or absorbs existing frameworks. Rather, Ψ_*f*_ connects aspects of each and extends selected cost-related aspects of them: it relates to cognitive load on the behavioral side, to allostatic load on the physiological side, and to variational complexity on the information-theoretic side, while adding explicit cross-modal latent modeling and dissociation predictions that these frameworks do not individually provide.

Relative to the expected value of control (EVC) framework (Shenhav et al., 2013, 2017), Ψ_*f*_ is closest to a transmodal control-cost extension. Both treat control as a cost-constrained process, and EVC's effort-cost term and Ψ_*f*_'s control-energy integral (Equation 3c) share formal structure. However, the two frameworks diverge on three points that generate distinguishable predictions:

First, EVC is typically fitted to single-task demand allocations (e.g., proportion congruent effects in Stroop), whereas Ψ_*f*_ accumulates *across* tasks and modalities within a session and predicts a *non-linear* saturation transition (ρ_*c*_) that standard EVC cost functions do not produce. A concrete illustration: under EVC, adding concurrent cognitive load to a task-switching paradigm increases effort cost approximately additively; under Ψ_*f*_, load accumulates toward a critical point beyond which switch cost accelerates non-linearly (Equation 7b). If segmented-regression analyses in H-NEURO-EXEC-01 show that the linear (additive) model fits as well as the changepoint model, the saturation component of Ψ_*f*_ is rejected (Table 5), effectively reducing Ψ_*f*_ to an EVC-compatible additive formulation.

**Table 5:** Alternative-model adjudication matrix (pre-registered decision rules).

Competing account	Core competing prediction	Ψ_f_ prediction	Decisive test	Predefined decision rule
Additive resource model	Switch cost rises approximately linearly with load	Changepoint/nonlinear acceleration near $\rho_{c}^{*}$	Compare linear vs changepoint models in H-NEURO-EXEC-01	If linear fit ≥ changepoint in primary ρ^*^ analysis, reject saturation component
Generic symptom-severity model	Clinical groups differ mainly by global severity	Disorder-specific dissociations (Table 2)	Phase-2 contrasts in §4.5	If dissociation contrasts fail, downgrade to transdiagnostic severity interpretation
Language-style/trait model	μ_sem_ mostly stable stylistic variance	Context-sensitive shifts + incremental validity	H72 context contrast (primary) + incremental validity over PHQ-9/GAD-7/affect controls (secondary)	If either fails, downgrade to exploratory; if both fail, remove linguistic probe from core model
Global-deficit account	Expansion markers add little structure beyond symptoms	Optional expansion markers may add mechanistic precision	Compare core vs expansion model fit in phase-3	If no incremental value, retain core chain and demote expansion markers to exploratory

Second, EVC does not natively generate *cross-domain dissociation* predictions of the kind central to the Ψ_*f*_ clinical mapping. For example, the joint prediction is that OCD patients show the highest μ_sem_ (linguistic domain) while PCI remains near the control range (neural domain) is a two-channel dissociation that EVC, as currently specified, does not address because it lacks a linguistic output channel and a cross-modal latent structure.

Third, EVC models control allocation within a trial or block but do not formally incorporate *slow-constraint dynamics* (Equation B1, Appendix B) governing habit formation, belief revision, and therapeutic change over weeks to months.

These distinctions do not make EVC wrong; they mark scope boundaries. If future EVC extensions incorporate cross-modal indicators, non-linear saturation, and slow dynamics, the two frameworks may converge. The present contribution is that Ψ_*f*_ provides this integration now in a testable form. This positioning is consistent with computational psychiatry goals of mechanistic yet clinically testable bridges (Huys et al., 2016).

### 6.3 Limitations and boundary conditions

Several important limitations must be acknowledged.

First, Ψ_*f*_ is a *latent construct* inferred from multiple indicators. No single measure is sufficient, and the convergent validity of the four proxy classes is an empirical claim that remains to be tested. If the structural equation model (§3.5) fails to support a single-factor solution, the framework requires fundamental revision.

Second, the modal verb probe (μ_sem_) may be *language-specific*. The inventory of modal verbs and their semantic valence varies across languages (Palmer, 2001), and the constraint/possibility distinction may not partition cleanly in all linguistic systems. Cross-linguistic validation with Mandarin Chinese, German, and Arabic modal systems is a necessary follow-up.

Third, distress and pain are *not* assumed to be generated solely by control cost. Equation 4 is a conditional hazard model; nociceptive injury, social threat, affective dysregulation, and appraisal shifts can all elevate distress even when *dΨ*_*f*_/*dt* is modest. This is why we model a hazard family with smoothing and appraisal terms rather than a one-to-one derivative identity.

Fourth, the ROS coupling hypothesis (Equation 9) requires *longitudinal data* to establish temporal precedence. A cross-sectional correlation between Ψ_*f*_ and ROS markers, while consistent with the model, does not establish directionality. Prospective designs and intervention studies (manipulating Ψ_*f*_ and measuring ROS change) are essential.

Fifth, the equations presented (Equations 3a−5, plus Equation B1 in Appendix B) use simplified functional forms. In particular, the control-cost quadratic form in Equation 3c, the KL form in Equation 3d, and its local Fisher approximation in Equation 3d' are modeling choices, not identifiability guarantees. The precise friction-cost shape (quadratic, piecewise, exponential, or sigmoidal) remains an empirical question.

Sixth, the framework is *silent on the hard problem of consciousness* in the Chalmersian sense (Chalmers, 1995). Ψ_*f*_ provides a measurable correlate of the cost of maintaining conscious states, but it does not explain *why* subjective experience accompanies high-friction processing. We view this as an appropriate scope limitation rather than a deficiency.

Seventh, every proxy proposed here is *indirect*. None of the behavioral, physiological, neural, or linguistic measures observes executive friction directly; each is a downstream correlate standing at some remove from the latent quantity, and each carries its own non-friction variance (task-switch cost reflects general processing speed, HRV reflects fitness and respiration, modal usage reflects register and education). This has two consequences for interpretation. It means construct validity cannot be settled by any single indicator and rests entirely on convergence, so the framework is only as strong as the cross-modal correlation structure in Section 3.5 turns out to be; if that structure is weak, the “friction” label may be over-interpreting a loose bundle of partially related measures. It also means effect sizes at the indicator level will understate the latent association, and that inferences about Ψ_*f*_ are conditional on the measurement model rather than read straight off the data. We regard demonstrating that these indirect proxies actually cohere, and discriminate Ψ_*f*_ from adjacent constructs, as the central empirical burden of the program rather than a settled premise.

Eighth, the disorder mappings overlap, and the boundaries between them are provisional. The clearest case is switching: OCD is framed as elevated switching viscosity with preserved maintenance, yet depression also impairs switching, and anxiety, rumination, and generalized psychomotor slowing can each mimic parts of the predicted signatures. The framework's disorder-specific claims therefore rest on multivariate *profiles* (Table 2) and on the paired dissociation contrasts of Section 4.5, not on any single proxy, and they are correspondingly vulnerable: if those contrasts fail to separate the conditions, the clinical mapping should be read as a transdiagnostic severity gradient rather than as a set of distinct disorder signatures. We consider this boundary ambiguity a real risk to the account, not a peripheral caveat.

Ninth, this article reports *no empirical data*. While we have specified protocols with sufficient detail for preregistration and replication, the entire framework remains at the stage of theoretical proposal until the validation program (§5.6) is executed.

### 6.4 Alternative models and decisive tests

The framework is designed to be partially rejectable rather than all-or-none. Key adjudication rules are:

- If linear models consistently outperform changepoint models, reject the saturation component (Equation 7b) while retaining additive control-cost formulations.
- If cross-modal convergence fails (between-domain correlations uniformly < 0.15), reject the single latent-factor claim and move to domain-specific modules.
- If μ_sem_ lacks either context sensitivity (primary construct-validity test) or incremental validity (secondary utility test), downgrade the linguistic probe to an exploratory indicator; if it lacks both, remove it from the core chain and retain behavioral-physiological modeling.
- If optional expansion markers (ROS/PCI/Fisher) do not add predictive value, retain the core chain and demote expansion markers to exploratory status.

These outcomes define explicit model-pruning pathways and prevent *post hoc* theory protection.

### 6.5 Translational potential

If empirically validated, the Ψ_*f*_ framework offers several translational applications.

#### 6.5.1 Transdiagnostic biomarker

The friction signature profiles (Table 2) could enable a biologically grounded, transdiagnostic classification of executive dysfunction that cuts across traditional categorical diagnoses.

#### 6.5.2 Passive linguistic monitoring

μ_sem_ can be extracted from clinical interview transcripts, therapy session recordings, or, with appropriate consent and ethical oversight, naturalistic text data. This enables longitudinal Ψ_*f*_ tracking without additional testing burden.

#### 6.5.3 Treatment response prediction

Therapeutic interventions can be modeled as either reducing Ψ_*f*_ (pharmacological approaches targeting the friction cost) or increasing *P*_sel_ (behavioral approaches expanding selection power capacity). The Ψ_*f*_ trajectory over treatment could serve as an early indicator of therapeutic response, potentially outperforming symptom-based monitoring.

#### 6.5.4 Intervention design

The distinction between Ψ_*f*_ reduction and *P*_sel_ expansion suggests that combination therapies (pairing pharmacological Ψ_*f*_ reduction with behavioral *P*_sel_ training) should show synergistic effects evidenced as follows:

$$\begin{array}{l} \Delta S_{\text{s}\text{y}\text{n}\text{c}}=\Delta d\cdot \left(-\Delta {\text{Ψ}}_{f}\right)>0\; \end{array}$$

where a positive synergy metric indicates genuine cross-domain recovery rather than compensation.

## 7 Conclusion

We have introduced executive friction (Ψ_*f*_) as a formally defined, cross-modally operationalizable construct that transforms the control cost of maintaining or switching an active state against baseline defaults into a testable framework for cognitive and clinical dynamics. The construct is grounded in core equations that specify friction accumulation (Equations 3a−3d'), the hazard function for subjective distress (Equation 4), the selection-budget inequality (Equation 5), and the dynamics of parameter evolution (Equation B1). We have operationalized Ψ_*f*_ through four convergent proxy classes (behavioral, physiological, neural, and linguistic), and demonstrated how the knowing–doing gap emerges as bandwidth saturation when friction cost exceeds available selection power.

The clinical mapping onto major depressive disorder, obsessive-compulsive disorder, and Parkinson's disease generates disorder-specific, falsifiable predictions (Table 2) that distinguish the Ψ_*f*_ account from generic executive dysfunction descriptions. Five preregistration-ready hypotheses with explicit falsification criteria and a three-phase validation roadmap provide the experimental architecture for systematic testing.

The knowing–doing gap is not merely a clinical curiosity but a window into the control-cost structure of agency. Every act of will, from suppressing a craving to overriding a prejudice to initiating a motor sequence, incurs executive friction. Understanding this cost, measuring it, and learning to modulate it may prove to be consequential for cognitive neuroscience, clinical science, and biomarker-driven translational work.

## Data Availability

The original contributions presented in the study are included in the article/supplementary material, further inquiries can be directed to the corresponding author/s.

## Appendix A. Optional symbol mapping and background citation

The core model in this study can be read entirely in standard control/predictive-processing language. For readers who prefer compact symbols, the optional shorthand is listed as follows:

- Π : candidate state/policy space
- *x*_act_: currently executed active state
- *z* : slow prior/habit/hyperprior constraints
- *Select*_θ_: resource-bounded selection or gating map
- Ψ_*f*_: latent control cost
- *P*_sel_: available control budget
- *S*_noise_: exogenous or environmental uncertainty load.

No commitment to any broader framework is required for evaluating this study's equations, hypotheses, or falsification logic; all claims are self-contained and independently testable. The present article uses the empirically neutral label “executive friction” throughout and treats this appendix as optional notation support rather than as a gateway to a larger philosophical system.

## Appendix B. Parameter dynamics equation (slow timescale)

The embodiment parameters θ evolve according to the following:

$$\begin{array}{l} \frac{d\theta }{dt}=\underset{\text{Learning}}{\underset{\underset{\;}{\underset{︸}{\;}}}{\eta \cdot A[\sigma ,\text{Target}]}}-\underset{\text{Friction}\;\text{descent}}{\underset{\underset{\;}{\underset{︸}{\;}}}{\delta \cdot \frac{\partial \Phi (\theta )}{\partial \theta }}}-\underset{\text{Homeostatic}\;\text{recoil}}{\underset{\underset{\;}{\underset{︸}{\;}}}{\kappa \cdot ({\text{Input}}_{\text{act}}-\text{Baseline})}} \end{array}$$ (10a)

where η, δ, *and κ* > 0, *A*[σ, Target] is the learning signal (error-driven adaptation), ∂Φ(θ)/∂θ is the gradient of the friction potential (driving parameters toward lower-friction configurations), and the third term captures homeostatic pressure to return to baseline. This equation governs the slow dynamics of habit formation, belief revision, and therapeutic change over weeks to months.

Equation (B1) is not tested in the current phase-1/2 validation roadmap because it requires longitudinal treatment-design data. It is included here for theoretical completeness and to motivate future intervention studies in which pharmacological Ψ_*f*_ reduction (modifying Φ) and behavioral *P*_sel_ training (modifying η or κ) can be modeled as distinct parameter-level interventions.
